# Quantum dots for bone tissue engineering

**DOI:** 10.1016/j.mtbio.2024.101167

**Published:** 2024-08-03

**Authors:** Ning Ding, Fengjin Zhou, Guangfeng Li, Hao Shen, Long Bai, Jiacan Su

**Affiliations:** a Organoid Research Center, Institute of Translational Medicine, Shanghai University, Shanghai, 200444, China; b Department of Orthopedics, Xinhua Hospital Affiliated to Shanghai Jiao Tong University School of Medicine, Shanghai, 200092, China; c National Center for Translational Medicine (Shanghai) SHU Branch, Shanghai University, Shanghai, 200444, China; d Wenzhou Institute of Shanghai University, Wenzhou, Zhejiang, China; e Department of Orthopaedics, Honghui Hospital, Xi'an Jiao Tong University, Xi'an, 710000, China; f Department of Orthopedics, Shanghai Zhongye Hospital, Shanghai, 200444, China

**Keywords:** Bone tissue engineering, Quantum dots, Bioimaging, Drug delivery, Artificial intelligence, Bone organoids

## Abstract

In confronting the global prevalence of bone-related disorders, bone tissue engineering (BTE) has developed into a critical discipline, seeking innovative materials to revolutionize treatment paradigms. Quantum dots (QDs), nanoscale semiconductor particles with tunable optical properties, are at the cutting edge of improving bone regeneration. This comprehensive review delves into the multifaceted roles that QDs play within the realm of BTE, emphasizing their potential to not only revolutionize imaging but also to osteogenesis, drug delivery, antimicrobial strategies and phototherapy. The customizable nature of QDs, attributed to their size-dependent optical and electronic properties, has been leveraged to develop precise imaging modalities, enabling the visualization of bone growth and scaffold integration at an unprecedented resolution. Their nanoscopic scale facilitates targeted drug delivery systems, ensuring the localized release of therapeutics. QDs also possess the potential to combat infections at bone defect sites, preventing and improving bacterial infections. Additionally, they can be used in phototherapy to stimulate important bone repair processes and work well with the immune system to improve the overall healing environment. In combination with current trendy artificial intelligence (AI) technology, the development of bone organoids can also be combined with QDs. While QDs demonstrate considerable promise in BTE, the transition from laboratory research to clinical application is fraught with challenges. Concerns regarding the biocompatibility, long-term stability of QDs within the biological environment, and the cost-effectiveness of their production pose significant hurdles to their clinical adoption. This review summarizes the potential of QDs in BTE and highlights the challenges that lie ahead. By overcoming these obstacles, more effective, efficient, and personalized bone regeneration strategies will emerge, offering new hope for patients suffering from debilitating bone diseases.

## Graphical abstract

**Figure:**
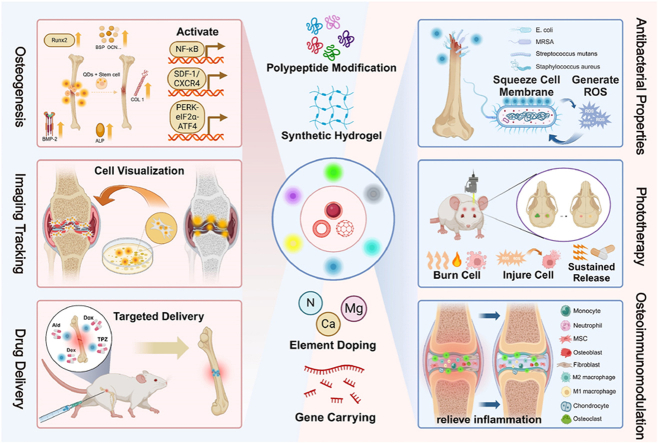

## 1 Introduction

Bone has a certain healing and regeneration ability [1], but some special diseases, such as segment bone defect, often lead to bone non-union, delayed healing or non-healing [2], which becomes a serious challenge in the field of bone repair. Autogenous bone transplantation is still the “gold standard” of clinical bone repair, but it inevitably leads to secondary surgical injury, serious donor site injury and other complications [3]. Allogeneic bone, as an alternative, also has a risk of immune rejection [4] and transmission of disease, and its osteogenic capacity and biological activity is usually lower than that of autologous bone [5]. With the advancement of research, the intervention of a series of bone tissue engineering (BTE) strategies starting from scaffold materials has brought light to the treatment of bone defects.

Scaffold materials can induce the formation of new bone tissue and eliminate the risks associated with bone transplantation [6]. Calcium phosphate, as a bioceramics, has excellent bone induction properties. Wei et al. designed a biphasic calcium phosphate bioceramic scaffold and combined it with intramedullary nails to stabilize the osteogenic microenvironment and achieve good results in the treatment of large segmental bone defects in goat femur models [7]. However, bioceramics themselves do not have sufficient mechanical properties and bone-inducing properties, which limits their clinical application. Some natural polymers, including alginate [8] and collagen [9], also exhibit remarkable biomineralization properties and possess the capability to promote cell attachment and proliferation. But they do not have sufficient mechanical properties and degradability. Therefore, at this stage, it is often used to construct multi-functional material scaffolds with materials such as bioceramics, which involves more complex biocompatibility issues. Moreover, synthetic polymers face the same challenge. Polyethylene Glycol (PEG) based nanocomposites show good biocompatibility and drug delivery ability in bone tissue regeneration, but the degradation rate, mechanical properties and cost are still not perfect [10]. Moreover, the distribution of cells after scaffold transplantation, whether and how cells proliferate and differentiate, and related mechanisms of action are also related to bone repair therapy. Therefore, bioactive materials capable of simultaneously offering real-time dynamic monitoring of cellular behavior and intervening in the key biological processes of bone repair hold significant promise in the field.

In 2023, quantum dot (QD) technology was awarded the Nobel Prize in Chemistry for its outstanding contributions to the fields of biomedicine and materials science. QDs are nanoscale particles comprised of elements from groups II-VI or III-V in the periodic table [11,12], typically exhibiting a particle size ranging between 1 and 10 nm. Due to its small size and quantum confinement effect [13], QDs exhibit unique fluorescence properties that are resistant to photobleaching. They can be adjusted in various ways to obtain the desired fluorescence color, and have great potential as fluorescent probes in biomedical fields. QDs, known for their structural stability, possess the remarkable capability to readily traverse cell membranes [14] and high specific surface area [15]. Research has revealed their resilience under diverse conditions, including fluctuations in pH levels, prolonged exposure to high levels of ultraviolet (UV) radiation, and oxidative environments [16]. They can also be coupled with different ligands so that they can load drugs through hydrophobic, non-covalent π-π stacking interactions and hydrogen bonds, showing certain advantages in drug delivery. Bone repair is a multifaceted and dynamic process involving cell migration, proliferation, differentiation and new bone formation. This process requires precise spatio-temporal control to ensure functional recovery of bone tissue [17]. The fluorescence properties of QDs can not only label various relevant cells during bone repair, accurately detect their behavior in the bone defect area, but also detect the release and distribution of drugs while they are delivered. The sulfur-containing carbon QDs (CQDs) synthesized by Zhu et al. exhibited robust stability in aqueous solutions and demonstrated outstanding compatibility and affinity with bone cells. Due to its low cytotoxicity, it is considered to have the potential to be used as a fluorescent probe to detect and distinguish cells associated with bone lesions, providing a new tool for the diagnosis and research of bone diseases [18]. Sheng et al. prepared graphene QDs (GQDs), whose surface carboxyl groups can undergo acylation reactions with the amino group of the anticancer drug cytidine. The amide bonds between them can be hydrolyzed in acidic media to obtain pH sensitive drug loaded GQDs, opening up a new path for intelligent drug delivery systems [19]. Relevant studies also found that QDs can participate in bone immune regulation and antibacterial [20,21], combined with phototherapy treatment of related bone diseases. It is clear that QDs has better properties than traditional materials and has broad application prospects in BTE, but there is a lack of systematic reviews in this field.

**Fig. 1:**
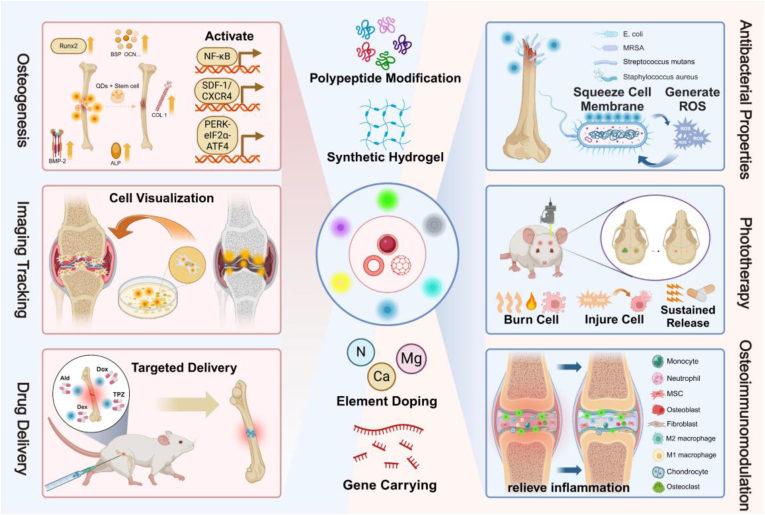
**The application of QDs to bone repair in biomedicine.** Multiple modifications of QDs and their application principles in osteogenesis, imaging tracking, drug delivery, antimicrobial properties, phototherapy, and osteoimmunomodulation.

In this review, we initially explored the development process and current applications of QDs in the field of nanotechnology. We systematically outlined the preparation methods and various types of QDs. Subsequently, we delved into the relevant applications of QDs in BTE, covering osteogenesis, imaging, delivery, antibacterial properties, phototherapy, and osteoimmunomodulation (Fig. 1). Finally, we pointed out the challenges faced by QDs in BTE, and proposed the future development direction of its combination with AI to construct bone organoids. This review offers comprehensive theoretical backing for the integration of emerging nanomaterials, QDs, into the realm of BTE, thus laying the foundation for pioneering advancements in this domain.

## 2 Overview of QD technology

Nanotechnology is one of the important directions of science and technology development in the 21st century. It creates nanomaterials with unique properties by manipulating materials at atomic, molecular and supramolecular scales. Most nanomaterials have extremely high specific surface areas, and their size can control the half-life and distribution. Their surface properties also play a role in their distribution in the body, and charge has been shown to be one of the determining elements that they internalize into their target cells [22]. Nanomaterials can also respond to changes in the external environment and integrate multiple functions to meet application requirements. Kumar et al. synthesized a silver hydroxyapatite nanoparticles that do not change hydroxyapatite biocompatibility and are more effective at preventing bacterial growth [23]. And some ome researchers have found that osteogenic exosomes have good therapeutic properties. In a study, injection of exosomes can alleviate the phenomenon of delayed fracture healing in CD9^−/−^ mice, in which the microRNAs contained play an important role in inducing osteogenesis and angiogenesis [24]. Despite the potential of nanomaterials in areas such as bone repair, there are still challenges, especially in terms of the inability to monitor material dynamics in vivo. Due to their small size, traditional imaging techniques are difficult to accurately track nanomaterials, and their distribution, metabolism, and long-term effects in the body are not fully understood. Future research needs to develop new technologies to monitor the in-vivo behavior of nanomaterials in real time to ensure their safety and effectiveness.

QDs are semiconductor crystals characterized by a particle size typically ranging from 1 to 10 nm, consisting of tens to hundreds of atoms. Due to the nanoscale size of QDs, electrons and electron holes are subject to quantum confinement effects [25]. Their energy levels become discrete, with tunable band structures and band gap widths, giving QDs unique optical, electrical and magnetic properties. For example, when the applied energy matches the energy level of QDs, they can emit bright and stable light, which is called QDs luminescence and has unlimited potential in display technology, biological imaging, lighting and other fields [26]. Since the early 1980s, many scholars have studied QDs due to their rich physical and chemical properties, forming many important cutting-edge technologies. Undoubtedly, QDs bring hope for cutting-edge technologies in nanotechnology. In recent years, more and more QDs have been applied to the field of medicine and pharmacy. This part mainly introduces the current development context, synthetic means, classification of QDs, as well as the existing applications in biomedicine and tissue engineering. By summarizing the advantages and limitations of QDs, we believe that it has a broad development prospect in BTE.

### 2.1 Chronological development of QDs

**Fig. 2:**
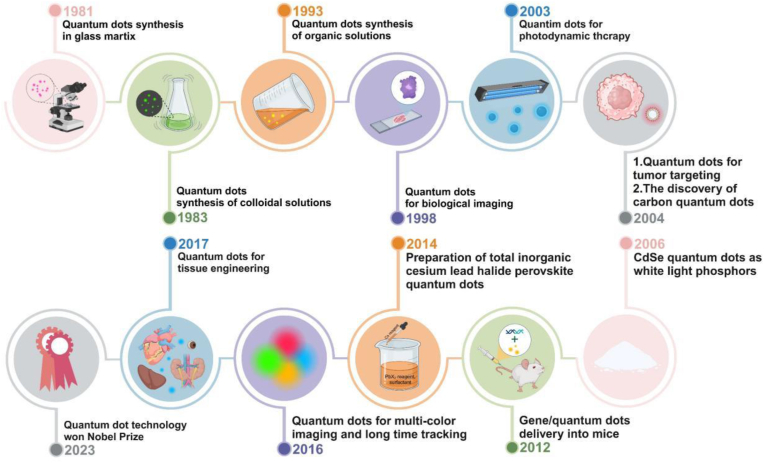
**Brief development history of QDs.** Major events related to QDs since their discovery.

In 2023, QDs technology in nanotechnology won the Nobel Prize in Chemistry, but QDs had already entered the vision of researchers before that. QDs were first discovered in 1981 by Alexey Ekimov and Louis Bruce in glass substrates and colloidal solutions [27]. They used cadmium telluride thin films in the experiment and successfully prepared cadmium telluride nanocrystals with a diameter of several nanometers through selective chemical synthesis methods [28]. As a result, it opened the prelude for scientists to study QDs. Fig. 2 shows the significant development events related to QDs from 1981 to 2024 (Fig. 2).

### 2.2 Classification of QDs

**Fig. 3:**
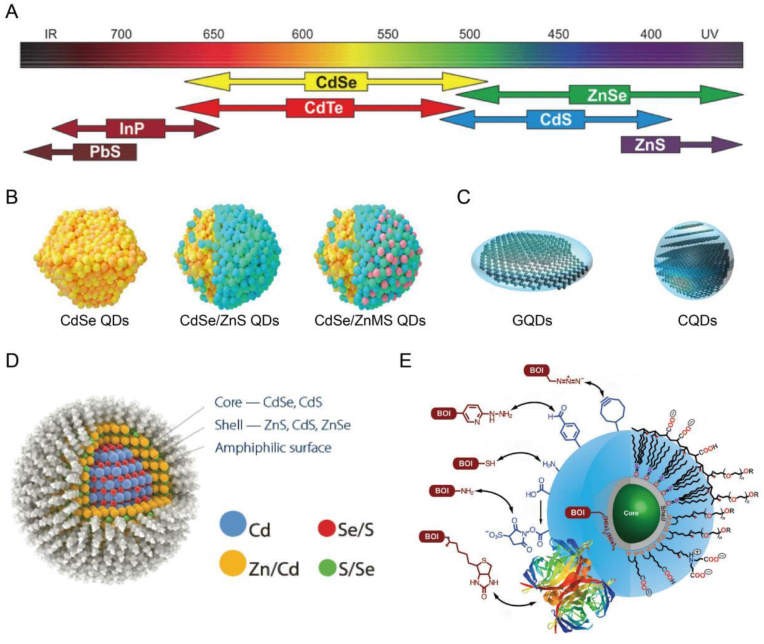
**Structure and modification of QDs.** A) Different types of QDs and their corresponding radiation. Reproduced and adapted with permission [29]. Copyright 2014, Tabriz University of Medical Sciences. B) CdSe QDs, CdSe/ZnS QDs and selectively integrated QDs models. Reproduced and adapted with permission [30]. Copyright 2020, Elsevier. C) Model diagram of CQDs and GQDs. Reproduced and adapted with permission [31]. Copyright 2023, Elsevier. D) The most common core-shell QDs model. Reproduced and adapted with permission [33]. Copyright 2016, Baqiyatallah University of Medical Sciences. E) Common interfacial chemical modifications and biological coupling of QDs. Reproduced and adapted with permission [26]. Copyright 2013, SAGE Publications Inc.

Different types of QDs [29] generally correspond to different radiation ranges (Fig. 3A). And QDs is generally spherical or quasi-spherical, but it is classified according to geometric shape, and there are tetrahedral QDs, cylindrical QDs and cubic QDs. According to the material can be divided into organic QDs and inorganic QDs. Semiconductor QDs in inorganic QDs are typically composed of group IV-IV elements (including PbS, PbSe), Group II-VI elements (including CdS, CdSe (Fig. 3B) [30], CdTe, ZnS), or Group III-V elements (including InP, InAs). In recent years, organic QDs that do not contain heavy metals such as Cd and Pb, such as CQDs [31], GQDs (Fig. 3C) and SiQDs, have attracted more and more attention. According to the classification of structural types, QDs can be divided into core QDs, core-shell QDs, alloy QDs and multilayer QDs, and the most common and popular is core-shell QDs (Fig. 3D) [32,33].

In addition to these simple types, more complex QDs can be obtained by introducing different surface modifiers or coatings on the QDs surface to change the properties and characteristics. Most QDs utilized in biological applications are synthesized in core-shell structures, making the outermost layer the primary site for modification by experts. Petryayeva et al. have made a systematic summary of the interfacial chemical modification and biological coupling of QDs (Fig. 3E) [26]. Surface modification mainly involves conjugating QDs to various biomolecules (such as proteins, peptides, or antibodies) to enhance their stability and specificity in the biological environment. For example, by treating with amphiphilic polymers or PEG, QDs can gain water solubility, reduce non-specific binding, and improve cycle time in complex biological environments [34]. Surface modifications can achieve covalent binding of QDs to bone repair related molecules by introducing specific functional groups, such as carboxylic acids, amines, or mercaptan groups. Biological targeting uses these surface modified functional groups to connect QDs with specific biomolecules to achieve bone tissue or cell targeting. For example, QDs can be targeted to bone defect areas using peptide sequences or antibodies that have a high affinity for bone morphogenetic proteins (BMPs). This targeting ability can not only improve the specificity of imaging, but also serve as a drug carrier to deliver therapeutic molecules directly to the injury site, thereby promoting bone tissue regeneration. We will introduce the details in the following. In short, different customization methods make QDs have more unique physical and chemical properties and biological activities, and are expected to become a new generation of nanoscale scaffolds.

### 2.3 Synthesis of QDs

The first significant milestone in the preparation of QDs occurred in 1983, when Bawendi and his team demonstrated how to rapidly mix chemical precursors through thermal injection to prepare unprecedented cadmium sulfide colloidal nanocrystals [35]. Since then, the preparation of QDs has entered the public's field of vision. Various preparation methods have been developed for QDs, which are broadly categorized into two types: top-down and bottom-up approaches [36]. Table 1 summarizes some of the main preparation methods.

**Table 1:** Synthesis method of QDs.

Type of Synthesis		Example	Ref.
Top-down	γ-Irradiation	CdCl_2_, Na_2_S_2_O_3_ and (CH_3_)_2_CHOH were mixed in distilled water, the silk fibers were added, the N_2_ was bubbled to remove O_2_. Then γ-ray irradiation was carried out. Finally the CdS QDs was obtained by washing with ethanol and water and drying.	[37]
	Molecular beam epitaxy	Monolayer lnAs are deposited on the surface of single crystal GaAs and subsequently overgrow with GaAs. Isolated InAs QDs were formed In GaAs matrix by cascade flow nucleation of Ga and In materials after adjusting growth conditions in situ by reflected high-energy electron diffraction.	[38]
	Ion implantation	In a setting of reduced temperatures and brief annealing durations, the synthesis of ZnO QDs within a silica matrix is achieved through a sequential process of zinc and fluorine ion injection, followed by a controlled heat treatment. The genesis of these ZnO QDs is attributed to the oxidation process, which is initiated by the introduction of fluorine ions and catalyzed by the presence of zinc nanoparticles.	[39]
	Oxidative cleavage	Small graphene oxide flakes undergo treatment with nitric acid to form small cut blocks. Subsequently, the surface is passivated with polyethylene glycol and reduced using hydrazine hydrate to obtain GQDs with small sizes.	[40]
	Electrochemical exfoliation	GQDsare synthesized via the electrochemical breakdown of carbon-based materials, including carbon nanotubes and graphite rods. This process involves the oxidation of water at the anode, leading to the generation of reactive species like hydroxyl and oxygen radicals. These highly reactive species are responsible for the fragmentation of the carbon precursor into GQDs.	[41]
	E-beam lithography	Aromatic molecules like anisole can undergo direct conversion into GQDs with nanostructures via cryogenic electron beam writing. The outcome of electron beam irradiation demonstrates consistent red fluorescence emission when stimulated by a 473 nm laser, with the capability to modify the photoluminescence intensity through the control of the electron beam irradiation dosage.	[42]
Bottom-up	High temperature	Water dispersible CdS QDs with emission wavelength of 510–650 nm were synthesized from cadmium chloride, thioureide and 3-mercaptopropionic acid by a simple one-pot non-injection hydrothermal route.	[43]
	Microwave assisted	In methanol solution containing polyethylpyrrolidone as stabilizer, HAuCl_4_ was rapidly reduced by microwave heating, and small spherical, monodisperse gold QDs were synthesized.	[44]
	Sol-gel methods	Solutions containing Cd and Se were grown at low temperatures to form wet gels due to heat treatment. It gives QDs of the size of 4–20 nm.	[45]
	Polyol methods	Diethylene glycol and the related metals zinc and cadmium were mixed with thiourea. To synthesize the nanoparticles, the acetate mixture nanoparticles were heated at 180 °C for 2h.	[46]

#### 2.3.1 The way of top-down

The top-down approach involves cutting large structural materials through physical or chemical methods, mainly including arc discharge, laser ablation, ultrasonic treatment, chemical ablation, and chemical oxidation [47]. The top-down method is widely used due to its easy availability of raw materials and high product yield. Although QDs synthesized by these methods have the advantages of high yield and easy access to raw materials, the cost is high and the process is complex. In BTE, it is necessary to pay attention to its particle size distribution, surface functionality and potential toxicity to ensure that the material can safely and effectively promote bone tissue repair and regeneration. Therefore, most top-down methods is not suitable for the synthesis of QDs applied in BTE.

#### 2.3.2 The way of bottom-up

The bottom-up pattern is to aggregate small molecule precursors into large-sized QDs through chemical reactions, mainly including water/solvent thermal method, template method, microwave-assisted method, and solid-phase method [48]. In recent years, people have chosen these more environmentally friendly methods. When synthesizing QDs in the field of BTE, considering the factors of biocompatibility, size control and production efficiency, I think water/solvent thermal method and template method are suitable methods. water/solvent thermal method maintains the biocompatibility of QDS by mild reaction conditions and non-toxic solvent, and can precisely control its size and crystallization, which meets the specific needs of BTE to some extent. By selecting specific template molecules, the template method accurately controls the morphology and bioaffinity of QDs, so that it exhibits excellent biological activity and targeting in bone repair. These methods not only take into account the precise control of material properties, but also comply with the principle of environmentally friendly synthesis, and promote the research and application of medical materials under sustainable development.

### 2.4 The current application status of QDs in the biomedical field

QDs, as a type of nanomaterial, have been widely used in many fields due to their unique optoelectronic properties and electronic effects. Based on the luminescent properties of QDs, the color gamut and saturation of the display screen can be improved, making the image more realistic and exquisite. This technology has been widely applied in electronic products such as televisions, mobile phones, and computer monitors [49]. QDs have adjustable band structures and excellent optoelectronic properties, which can be used to prepare high-efficiency solar cells [50], high brightness light-emitting diodes [51], and high sensitivity optoelectronic sensors [52]. Meanwhile, QDs can absorb visible light, convert it into chemical energy, and drive reactions such as organic degradation and water splitting, so they play a certain role in improving energy storage [53]. Using QDs as quantum bits to store and transmit quantum information can also achieve the popularization of quantum computing and quantum communication [54].

**Fig. 4:**
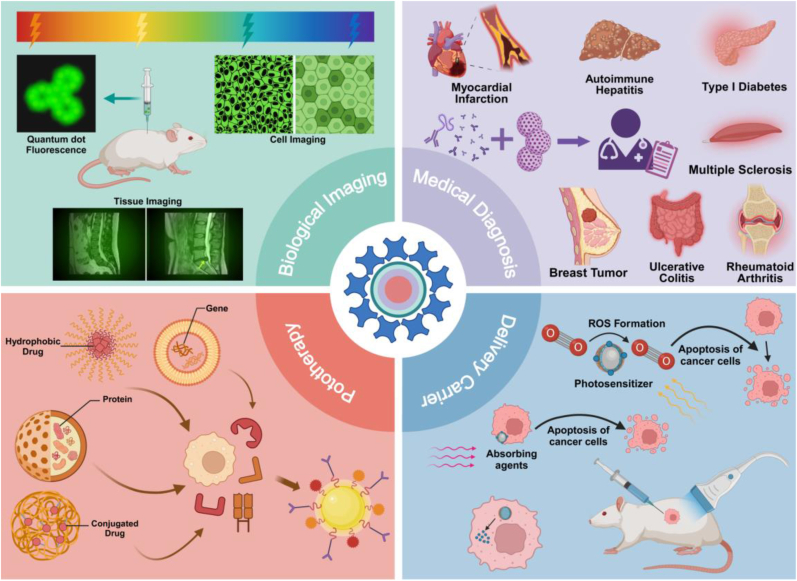
**QDs in Biomedicine: Imaging, Diagnosis, and Therapy.** Due to its unique optical and chemical properties, QDs is widely used in biological imaging, medical diagnosis, chemical phototherapy and as a drug carrier, and has diversified applications in the biomedical field.

QDs has become an ideal biomarker for cell and tissue imaging due to its high brightness, light stability, and tunable emission wavelength. This makes it have a broad development prospect in the field of biological imaging. At the same time, QDs has high sensitivity and specificity in the detection of disease markers, which is helpful for early diagnosis and disease monitoring. QDs can also produce reactive oxygen species under light excitation, which can be used to kill cancer cells or inhibit pathogens, providing new possibilities for non-invasive treatments. In addition, QDs, as a porous nanomaterial, can deliver drugs, using its size and surface modification properties to enable targeted drug delivery. At present, the application of QDs in the biomedical field is mainly concentrated in four aspects: biological imaging, medical diagnosis, delivery and chemical phototherapy (Fig. 4). The following is a brief introduction to the cutting-edge research of QDs in popular research areas.

#### 2.4.1 QDs for biological imaging

Fluorescent cell imaging is a biomedical application technology. As a nanomaterial with a large two-photon cross-section [55], QDs can be irradiated with near-infrared (NIR) light using nanoparticles emitting wavelengths of approximately 650–900 nm, where water and tissue absorption are very weak, making deep tissue imaging possible and significantly improving imaging [56].

For example, the combination of QDs and MRI reagents such as gadolinium increases the imaging signal by nearly 40 times [57], helping to detect cell apoptosis and provide information at the cellular level, thereby detecting whether anti-tumor therapy kills cancer cells. In vitro studies, a variety of cancerous cell lines, including MCF-7 [58], HeLa [59], and C6 gliomas [60], have been effectively visualized with the application of CQDs. In recent years, researchers have also used cesium lead bromide QDs as scintillators for X-ray detection, in order to create safe and effective QDs for cancer detection in vivo [61]. Leveraging the fluorescent tagging capacity of QDs, an increasing number of investigators are employing QDs to investigate intricate relationships within the immune system. For instance, CdSe/CdZnS QDs have been utilized to examine the process of antigen internalization by dendritic cells [62]. In a study, CQDs based tumor detection probes were combined with aspartic acid to target brain tumors and observe the expression of epidermal growth factor receptor (EGFR) in human brain tumor cells [63,64]. In the surgical treatment of brain tumors, QDs can be swallowed by macrophages via intravenous injection and brought to the brain tumor area, thus achieving optical labeling of the tumor. This macrophage-mediated delivery provides a new approach to preoperative tumor labeling that helps surgeons get real-time optical feedback during excisions and biopsies. In addition, QDs' bioimaging capabilities and targeting through attachment of specific ligands make it a powerful tool for penetrating the blood-brain barrier and treating diseases of the central nervous system, especially brain cancer [65,66].

#### 2.4.2 QDs for medical diagnosis

QDs is not only limited to fluorescence imaging, but also can be used for multi-modal imaging such as magnetic resonance imaging and photoacoustic imaging, making it play an important role in the field of medical diagnosis.

Bhatnagar et al. employed amine-functionalized GQDs conjugated with anti-cardiac Troponin I (anti-cTnI) to create the anti-cTnI/afGQDs nanoprobes. Frster Resonance Energy Transfer between graphene and couplings can detect cardiac marker in blood within 10 min, becoming an early detection pathway for myocardial infarction [67]. Xing et al. have crafted a molecular nanoprobe that is cell-specific and dually functional, harnessing the properties of QDs and paramagnetism to facilitate both in vivo magnetic resonance imaging for diagnostic purposes and subsequent biopsy to reevaluate colon cancer [68]. Wang et al. successfully combined magnetic nanoparticles with surface coupled pancytokeratin antibodies and QDs with surface coupled Lunx and SP-A antibodies to detect and diagnose circulating tumor cells in patients with non-small cell lung cancer, providing an effective tool for early diagnosis and recurrence prevention [69]. Indeed, there are numerous examples of QDs being utilized in various applications. For example, QDs that are encapsulated with polyacrylate shells and conjugated with antibodies have been utilized for the detection of breast cancer. In a parallel application, QDs associated with antibodies have been implemented for the identification of respiratory syncytial virus infections [70].

#### 2.4.3 QDs for delivery carrier

In recent years, the research trend of building delivery systems for nanoparticles is increasing [36]. The fluorescence properties of QDs encourage real-time tracking and allow researchers to map the drug distribution process clearly, attracting the attention of scientists. Some in vivo studies have shown that QDs can achieve stable circulation in the body after intravenous administration and lasts longer in major organs such as the kidneys and liver. This prolonged circulation ensures the stability of the drug before reaching its intended target site [71,72]. Moreover, evidence suggests that QDs, possessing cell-targeting capabilities as a novel drug delivery system, can exploit acidic environments to facilitate the release of therapeutic drugs from delivery vectors [73]. This undoubtedly makes QDs a new type of delivery system.

Different types of QDs have different delivery potential. A CdSe QDs carrier for delivering anti-cancer drug Dox has been developed. In experiments using CdSe/CdS/ZnS QDs to deliver Dox to rat alveolar macrophages, researchers found that QDs drug complexes can reduce inflammation and improve targeted delivery levels [74]. In a study, 5-fluorouracil is encapsulated onto Mn ZnS QDs embedded in chitosan biopolymers and conjugated with folate to augment its targeting specificity towards folate receptors expressed in malignant tissues [75]. However, the delivery ability of QDs is not limited to delivering drugs, they are also used to deliver proteins or genes [76]. The positively charged amino group on the surface of polyethyleneimine-functionalized CQDs (PEI-CQDs) can make DNA aggregate and transfer through electrostatic action [77]. Glycine proline glutamate peptide is believed to enhance impaired memory and learning abilities in Alzheimer's disease patients. GQDs bind to this neuroprotective peptide by preventing β-amyloid protein aggregation improved the effectiveness of treatment and significantly improved the condition of APP/PS1 transgenic mice in the experiment [78]. It can be seen that QDs, as a new type of delivery system, have broad development prospects.

#### 2.4.4 QDs for chemical phototherapy

In chemical phototherapy, QDs can act as a photosensitizer (PS), absorbing specific wavelengths of light energy and converting it into reactive oxygen species or other forms of energy to destroy tumor cells or diseased tissues. Because its size can be regulated, selective absorption of specific wavelengths of light can be achieved, thereby improving treatment effectiveness and reducing damage to surrounding normal tissue.

Huang et al. prepared BPQDs@NH hydrogel by encapsulating black phosphorus (BP) QDs in hydrogel. Under NIR irradiation, hydrogels can produce reactive oxygen species (ROS), glutathione and other substances to destroy cell membranes, realizing efficient sterilization stimulated by light therapy. In addition, animal experiments show that BPQDs@NH can not only modulate the expression of endothelial growth factor but regulate basic fibroblast growth factor, reducing the inflammatory response on wound surface and promoting wound healing [79]. Wu et al. developed a multifunctional nanoprobe (H–MnO_2_/DOX/BPQDs) to achieve synergistic chemical phototherapy. Experiments showed that BPQDs was activated under a single 630 nm light irradiation, generating intracellular ROS for photodynamic therapy (PDT). And BPQDs showed high photothermal therapy (PTT) effect under a single irradiation of 808 nm. BPQDs can be combined with DOX to achieve synergistic treatment effect [80].

## 3 QDs applied to the bone tissue engineering

Stem cell-based therapies are an important therapeutic approach [81]. And in order to solve the problems of bone graft technology, we need to provide a supportive extracellular microenvironment, stimulating stem cells to drive tissue regeneration and forming a support platform for transplantation of cells or recruitment and retention of endogenous cells [82]. Therefore, cell-based osteogenic testing is an important aspect of evaluating bone repair. Many targeted materials are widely used, such as bioceramics containing hydroxyapatite or calcium phosphate [83], some natural polymers [84,85], synthetic polymers [86] and hydrogels [87]. However, these materials are excellent at promoting bone regeneration and repair, but cannot enable real-time dynamic monitoring in vivo and in vitro. Real-time dynamic monitoring plays a crucial role in the bone repair process because it can better understand the degradation behavior of biomaterials in vivo, the formation of new bone, and the progress of the entire repair process, which is important for optimizing treatment options, preventing potential complications, and improving treatment success.

In contrast to conventional ceramics and polymers [88], the hydroxyl and amino groups on the surface of QDs can directly bind with BMP-2, SR-7, PS-11, IP-3, CK-23 and other components to promote the healing and regeneration of bone tissue [89]. QDs have the potential to induce cell death in tumor cells, including bone cancer cells [90]. QDs themselves exhibit excellent antibacterial properties, making them viable materials for preventing or treating common infections following bone defect repair [91]. Most importantly, QDs have unique optical properties that can track cell fate related to bone tissue regeneration and biodegradation of scaffold materials [[92], [93], [94]], as well as the potential for dynamic monitoring. Therefore, QDs can be well applied to BTE to promote its development. However, the current research progress regarding the combination of QDs and BTE remains relatively slow.

In addition, bone repair has an inseparable relationship with angiogenesis and immune regulation. Considering the various aspects involved in bone repair and the application of QDs in biomedicine, Next we start with osteogenesis, Imaging and fluorescence tracing, delivery, antibacterial properties, phototherapy, and osteoimmunomodulation.

### 3.1 Osteogenesis in BTE

Mesenchymal stem cells (MSCs) possess the remarkable ability to self-renew while preserving their pluripotency. This unique property allows them to differentiate into different cell types, including but not limited to osteoblasts and chondrocytes [[95], [96], [97]]. Research has revealed that the interaction between nanoparticles and MSCs can influence the self-renewal and differentiation capabilities of MSCs [98,99]. Based on this characteristic, MSCs play a crucial role in bone tissue repair and regeneration. In the past few years, there has been sufficient literature to prove that QDs can promote bone tissue regeneration.

Amino-β-cyclodextrin (β-CD) and Cys-Lys-Arg-Arg-Gly-Asp (CKKRGD) peptides were conjugated to CdTe/ZnS QDs to fabricate osteogenetic nanoparticles. β-CD encapsulates dexamethasone (DEX), while the polypeptide facilitates the delivery of small interfering RNA (siRNA) into the cell. RGD-β-CD-QDs transported Dex and siRNA simultaneously, leading to a significant improvement in the osteogenic differentiation rate and efficacy of hMSCs in both in vitro culture and in vivo environments. The results demonstrated that this nanocomposite exhibited high peroxisome proliferator-activated receptor γ (PPAR γ) silencing efficiency, which increased the expression of the osteogenic marker gene Runt-related transcription factor 2 (Runx2), thereby effectively promoting osteogenic differentiation of hMSCs. At the same time, it is worth noting that QDs was tracked in vivo for a long period of up to 3 weeks, providing certain evidence for its adaptability to bone growth cycle [100]. Huang et al. found that QDs, especially Ag_2_S QDs, improve the differentiation efficiency of neural stem cells through synergistic regulation of Wnt/β-catenin and RA signaling pathways. However, Wnt signaling can promote the differentiation of MSCs into osteoblasts by stabilizing β-catenin and activating its downstream genes, while RA promotes the proliferation and maturation of osteoblasts through its nuclear receptors, enhancing bone formation. The synergistic effect of these two signaling pathways can undoubtedly enlighten relevant studies in BTE and provide important regulatory targets for the treatment of related diseases. In addition, QDs showed good biocompatibility during transfection, which is essential for the long-term survival and function of transplanted stem cells in vivo, providing a strong proof of its position in the field of BTE [101]. Zinc (Zn) plays a vital role in bone formation, serving as a crucial medium for mineralization. Zheng et al. coated ZnO QDs onto bioactive glass nanoparticles (BGN) through electrostatic interactions. The alkaline phosphatase (ALP) activity measurement results showed that ZnO BGN can maintain biocompatibility with hMSCs and and exhibits pH sensitive zinc ion release properties, effectively improving the osteogenic differentiation of hMSCs [91]. BP is a novel material that can provide the final degradation product phosphate, which helps regulate bone mineralization and resorption. Xu et al. doped BP QDs into Oligo Poly Fumarate (OPF) to fabricate nanocomposite hydrogels. BP QDs enhanced the proliferation, distribution, proliferation and differentiation of mouse embryonic osteoblast MC3T3 on OPF hydrogel, and also increased the development of vascular protein and f-actin, thus improving the osteogenic ability of this hydrogel [102]. Moreover, selenium (Se) has been found to enhance immune surveillance, regulate the proliferation and differentiation of BMSCs, and protect BMSCs from damage induced by oxidative stress. Li and colleagues employed SeQDs to create Se@SiO_2_ nanocomposites, which were then enveloped with a layer of polyvinylpyrrolidone (PVP). This composite underwent a hot water etching process to produce a porous structure of Se@SiO_2_ nanocomposites. The findings from their study suggest that these materials are capable of enhancing the migration of BMSCs, diminishing the production of intracellular ROS, and shielding BMSCs from apoptosis triggered by H_2_O_2_. Furthermore, the Se@SiO_2_ nanocomposites were found to stimulate osteogenic differentiation and expedite the healing of fractures, with the mechanism of action being associated with the activation of the SDF-1/CXCR4 signaling pathway. This highlights the potential of these nanocomposite materials as promising candidates for BTE and regenerative medicine applications [103]. The research based on different QDs varies, but it is worth noting that some QDs themselves have significant toxicity and have inhibitory effects on the osteogenesis of MSCs. CdSe/ZnS QDs were introduced into MSCs via liposome-mediated transfection. The experimental group exhibited higher ALP activity compared to the control group without QDs. Further analysis by reverse transcription polymerase chain reaction (RT qPCR) revealed significant inhibition of the expression of osteopontin and osteocalcin, two osteogenic markers [104]. Simultaneously, the mRNA and protein expression of type II collagen (Col 2) and oligosaccharides in chondrocytes were significantly inhibited, suggesting that the chondrogenic differentiation of BMSCs is affected by QDs. So, finding safe and non-toxic QDs is crucial.

**Fig. 5:**
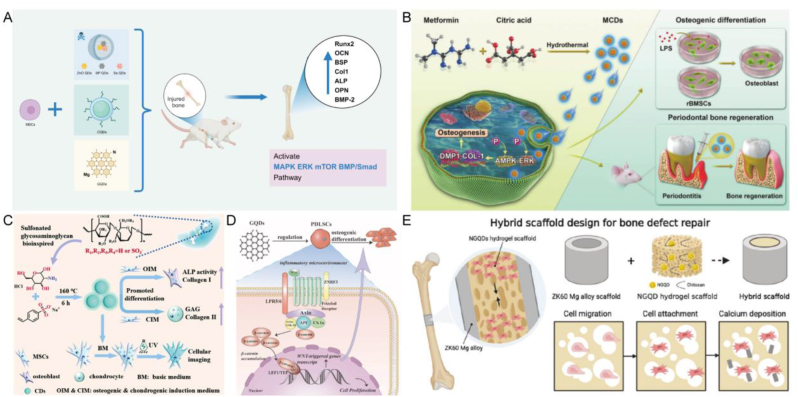
**QDs for osteogenesis in BTE.** A) A rough sketch of QDs combining with MSCs to promote bone formation. B) The synthesis of metformin-coated CQDs and their mechanism in promoting periodontal bone regeneration. Reproduced and adapted with permission [113]. Copyright 2021, John Wiley and Sons Ltd. C) The preparation process of sulfonated glycosaminoglycan bioinspired CQDs for effective cell labeling and promotion of mesenchymal stem cell differentiation. Reproduced and adapted with permission [111]. Copyright 2020, Royal Society of Chemistry. D) Effect of GQDs on proliferation of PDLSCs in simulated pro-inflammatory environment medium and standard medium. Reproduced and adapted with permission [114]. Copyright 2023, BioMed Central. E) Construct composite scaffolds by binding of N-GQDs to Mg for bone defect repair. Reproduced and adapted with permission [115]. Copyright 2023, American Chemical Society.

CQDs have attracted widespread attention because of their excellent biocompatibility and low cytotoxicity [105]. CQDs can up-regulate the expression levels of Runx2, osteocalcin (OCN), bone sialoprotein (BSP) and other osteoblast-specific gene markers, and promote effective osteogenic differentiation in the ROS-mediated MAPK pathway [106]. Guo et al. synthesized CQDs through hydrothermal method, and supplementing CQDs can upregulate the mRNA expression of more osteogenic related genes, such as fibrous collagen, ALP, osteopontin (OPN), BSP, and BMP-2, to promote the osteogenesis of BMSCs [107]. Jin et al. prepared ascorbic acid CQDs using microwave-assisted pyrolysis method. Quantitative analysis of Alizarin Red S (ARS) staining showed that compared with the control group and osteogenic induction medium group, the CQDs group had higher calcium deposition and could form new bone in the defect area, enhancing bone regeneration in vivo. Furthermore, CQDs have been shown to induce endoplasmic reticulum stress in preosteoblasts by elevating intracellular calcium levels. This activation subsequently triggers the PERK-eIF2α-ATF4 signaling pathway, leading to the upregulation of the transcription factor ATF4. Consequently, this cascade results in increased expression of bone-related factors such as BSP and OCN [108]. Bu et al. also developed ascorbic acid PEI-CQDs, which carries miR-2861 and plays a role in osteogenic therapy. CQDs can be effectively internalized into BMSCs through the endocytic pathway mediated by grid proteins, and miR-2861 can be delivered to cells, synergistically promoting in vitro osteogenic differentiation and in vivo bone regeneration [109]. Wu et al. synthesized a novel calcium phosphorus co doped CQDs for bone regeneration using ethanolamine phosphate and calcium gluconate as precursors. Ca/P-CQDs with good biocompatibility quickly enter the cytoplasm through endocytosis. Compared with the exposed CQDs group, the mineralization of MC3T3-E1 osteoblasts treated with Ca/P-CDs group was significantly increased, and the expression of bone differentiation genes was also significantly upregulated. Later in the experiment, Ca/P-CQDs were mixed with temperature sensitive hydrogels to achieve in situ administration of drugs to treat skull defects, which verified its ability to repair bone damage in vivo [110]. Cai et al. prepared sulfonated glycosaminoglycan biomimetic CQDs to achieve differentiation of MSCs. The determination of ALP activity and Col 1 expression showed that rBMSCs cultured in osteogenic induction medium with CQDs exhibited significant osteogenic differentiation, and mineral deposition had a significant promoting effect. Furthermore, the impact of CQDs on cartilage induction was investigated. The findings demonstrated a significant increase in the expressions of glycosaminoglycan and Col 2, directly indicating the promotive effect of CQDs on cartilage differentiation. Subsequent studies have revealed that CQDs activate a variety of signaling pathways and stimulate both osteogenesis and cartilage differentiation without compromising normal cell function (Fig. 5C) [111]. CQDs can also be used in combination with peptides. Gogoi et al. prepared a biopolymer by coupling CQDs with four different peptides. The experimental results showed that the material had a strong effect on osteoblasts, enhanced mineralization, and showed the ability of heterotopic bone formation [112]. In addition, Ren et al. prepared CQDs of metformin and found that they could effectively enhance the activity of ALP in rBMSCs, increase the expression of Runx2, Sp7, Col 1 and dentin matrix protein 1 (DMP1), and increase the formation of calcium deposition nodules. It was also found that the QDs was induced by the EPK/AMPK signaling pathway(Fig. 5B) [113].

As another major category of carbon based QDs, GQDs are also a current research hotspot. In 2008, Bonomalianko and Geim developed GQDs [116], which not only have the advantages of general QDs, but also have high quantum yields higher than CQDs, making them widely applicable in various biomedical fields. Studies have shown that GQDs do not affect the proliferation of MSCs at appropriate concentrations, and can be absorbed by MSCs with concentration dependent self-renewal. Regarding osteogenic differentiation, MSCs co-incubated with GQDs exhibited increased presence of osteogenic markers under microscopic observation. Additionally, the mRNA expression was concentration and time dependent, suggesting the promotion of osteogenic differentiation in MSCs. At the protein level, the expression of OPN and OCN also increased with the increase of GQDs concentration, consistent with the results at the mRNA level [117]. Geng et al. identified that the surface charge of nanoscale materials has the capacity to affect cellular responses. In their findings, they determined that negatively charged GQDs are capable of triggering the bone BMP/Smad signaling cascade. This activation initiates an increase in the expression of various genes and proteins associated with osteogenesis. Consequently, this leads to a marked improvement in the osteogenic differentiation process of hMSCs. Then, the negatively charged GQDs were mixed into GelMA hydrogel to construct a scaffold for the skull defect model of mice in the presence of photoinitiator. It was observed that the scaffold effectively induced the healing and regeneration of the bone defect [118]. Magnesium (Mg) is widely recognized for its close association with mineralization. Wong et al. devised a metal composite scaffold incorporating N-GQD hydrogel. This novel scaffold has demonstrated efficacy in promoting uniform bone growth. Moreover, new bone can be directed within the N-GQD hydrogel channels, leading to enhanced mineral density (Fig. 5D) [115]. At the same time, GQDs can also promote osteogenesis in the inflammatory microenvironment. Gao et al. studied the effect of GQDs on in vitro proliferation and osteogenic differentiation of periodontal ligament stem cells (PDLSCs). The findings revealed that in an inflammatory environment, the mRNA expression levels of osteogenic markers associated with GQDs were significantly elevated, accompanied by an increased number of mineralized nodules (Fig. 5E) [114].

From this, it can be seen that QDs have a certain effect in promoting osteogenesis, mainly reflected in upregulating the expression of osteogenic related genes. Different types of QDs can exhibit excellent performance, and due to their different surface characteristics, they can combine with different materials, thereby expanding and promoting the field of osteogenesis. However, based on current research, due to biological toxicity, the types of QDs that can promote osteogenesis are single and mainly concentrated in carbon based QDs. In the future, we are expected to develop more green, safe, and non-toxic QDs, or combine them with more types of biomaterials to promote osteogenesis.

### 3.2 Imaging and fluorescence tracing in BTE

In BTE, the progress of scaffold materials in imaging, labeling, and tracking depends on their low toxicity, high sensitivity, high resolution, and stability. Conventional fluorescent nanomaterials, such as those based on rare metals and polymer nanoprobes, may exhibit cytotoxicity and potentially hinder osteogenic differentiation, posing adverse effects in biomedical applications. Therefore, finding a non-invasive, real-time, and effective tracking method for regulating osteogenic differentiation is crucial for the occurrence and development of BTE. QDs have the potential to enhance the sensitivity and resolution of bioimaging even at lower analyte concentrations. Through meticulous control of their size and chemical composition, the emitted color of QDs can be precisely tailored to different wavelengths of light, thus laying the groundwork for their initial applications in BTE, real-time labeling imaging, and tracking.

**Fig. 6:**
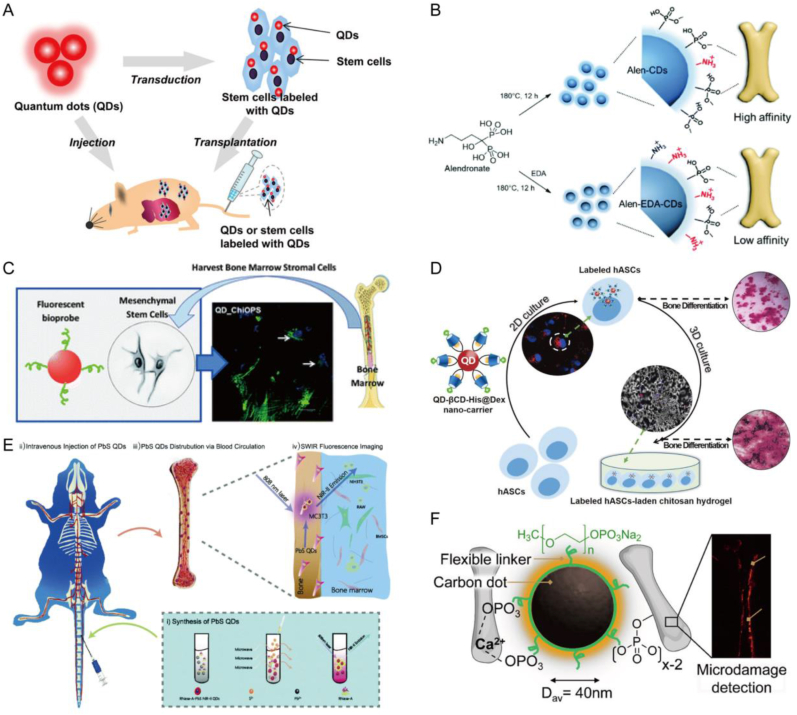
**QDs for imaging and fluorescence tracing in BTE.** A) Overview of QDs for in vivo fluorescence imaging and diagnosis of stem cells. Reproduced and adapted with permission [121]. Copyright 2017, American Chemical Society. B) Synthesis of Alen-CDs and Alen-EDA-CDs and schematic diagram of their bone affinity. Reproduced and adapted with permission [125]. Copyright 2019, Royal Society of Chemistry. C) Nanohybrid system constructed based on QDs with fluorescent core for labeling and imaging of bone marrow stromal cells. Reproduced and adapted with permission [124]. Copyright 2014, Royal Society of Chemistry. D) QD-βCD-His was demonstrated in 2D culture and 3D hydrogel scaffolds to label and track MSCs and their differentiation into bone tissue. Reproduced and adapted with permission [126]. Copyright 2020, Future Medicine Ltd. E) Synthesis of PbS QDs and its imaging under SWIR fluorescence after intravenous injection. Reproduced and adapted with permission [92]. Copyright 2020, Royal Society of Chemistry. F) Monodentate ligands bind to the CD surface to chelate Ca^2+^ exposed at the site of microcracks, and are used for the detection of bone microcracks by CD fluorescence. Reproduced and adapted with permission [127]. Copyright 2018, American Chemical Society.

Che et al. have successfully engineered a bone-specific imaging contrast agent, shortwave infrared QDs (SWIR QDs). Upon injection, these QDs circulate through the blood and identify major bone structures in mice, including but not limited to the skull, limbs, and spine. QDs have highly specific interactions with MC3T3-E1 cells, which explains the mechanism behind this bone imaging. This non-radioactive SWIR QD-guided real-time bone imaging holds promise for advancing comprehensive in vivo bone research and laying the groundwork for early diagnosis of bone diseases (Fig. 6E) [92]. Huang et al. is committed to using Ag_2_S QDs to achieve long-term tracking and imaging of the position, survival and osteogenic differentiation of transplanted hMSCs in vivo. These QDs showed excellent biocompatibility and stability in fluorescence imaging in the second near infrared window (NIR-II), did not cause oxidative stress, apoptosis, or DNA damage to cells, and had no negative effects on the migration, proliferation, and differentiation of hMSCs. By binding to the red firefly luciferase (RFLuc) and Gaussia luciferase (GLuc) reporter genes, Ag_2_S QDs not only labeled hMSCs, but also effectively indicated their survival status and osteogenic differentiation process. In addition, the study also found that immunosuppression and the use of BMP-2 can significantly improve the survival rate and osteogenic differentiation ability of hMSCs, further confirming the application potential of QDs in promoting tissue regeneration and improving the effectiveness of stem cell therapy [119]. Clinical trials using fluorescent nanoparticles have begun in the United States [120]. Indeed, a study has demonstrated that QDs offer high-resolution imaging capabilities at the cellular level. Leveraging fluorescence imaging techniques in vivo, QDs have the potential to facilitate stem cell transplantation in regenerative medicine (Fig. 6A) [121]. However, as a new type of nanomaterial, QDs in BTE fluorescence imaging is still in its nascent stages and has certain drawbacks. For example, the fate of BMSCs after intra-articular injection is not yet clear. Given the histological visualization of QDs-labeled BMSCs, Grady et al. used QDs to label equine MSCs, inject them into normal and osteoarthritic joints, to trace the fate of the MSCs after intra joint injection. Histological analysis revealed the presence of QD-MSCs in both synovial tissue and articular cartilage one week after transplantation. Importantly, in vitro experiments demonstrated that QD labeling did not adversely affect the proliferation, immunophenotype, or differentiation ability of MSCs. QD persisted for up to 8 weeks in non-proliferating cells, but in proliferating cells, QDs fluorescence essentially disappeared after two passes. Therefore, under the condition of cell proliferation, QDs is not suitable as a marker for long-term tracking of MSCs [122]. In addition to the aforementioned issues, QDs may also have certain toxicity to organisms. Research has shown that the introduction of cadmium selenide/zinc sulfide QDs into hBMSCs, although internalized QDs have been found in the perinuclear region and remain in multiple cell channels, significantly inhibits the expression of type Col 2 when inducing cartilage formation, interfering with the cartilage differentiation of MSCs [123]. However, due to the hydrophobicity of QDs, a study has functionalized them by covering their surfaces with biocompatible and water-soluble organic chitosan peptides, *o*-phospho-l-serine. Cadmium sulfide QDs directly coupled with chitosan *o*-phosphate serine peptide biocouples did not show significant cytotoxicity. The experiment was able to verify the efficiency of fluorescence labeling and the internalization of biological nanoprobes (Fig. 6C) [124]. In other words, this QDs can promote the absorption of conjugated cells and prolong the fluorescence intensity of cells. From this perspective, what kind of QDs to use and how to modify and coordinate these QDs have become the focus of researchers when studying their imaging tracking applications in BTE.

A fundamental issue with labeling MSCs with QD is cell compatibility during long-term and proliferative processes. To confront this issue, the prevalent approach is the application of a functional layer on the exterior surface of QDs, incorporating chemical moieties like carboxylate and a range of organic compounds. This modification enables QDs to couple with peptides or proteins, enabling instantaneous visualization and detailed microscopic examination. Shah et al. used RGD (Arg-Gly-Asp) conjugated QDs to label hMSCs and observed the performance of these labeled stem cells in the process of self-replication and differentiation into osteoblasts and chondrocytes. It was found that QDs-labeled hMSCs maintained similar activity and proliferation ability to unlabeled cells during the 22-day culture period, and QDs was mainly absorbed in the cytoplasm without entering the nucleus. During differentiation, QDs-labeled hMSCs were able to normally express specific markers associated with differentiation, suggesting that QDs does not interfere with normal stem cell differentiation. QDs can be used as an effective long-term cell labeling tool to further understand the role of stem cells in bone tissue regeneration [128]. BMP-2 is a crucial growth factor responsible for driving osteogenesis. However, the mechanisms governing the transport and absorption of BMP-2 into cells during disease progression remain unclear. In a recent study, researchers constructed a BMP-2-QDs conjugate. This innovative approach aimed to visualize the BMP-2 analog within the cellular environment and assess its stability and biological activity over time by binding to BMP receptors (BMPRs). The findings suggest that the BMP-2-QDs conjugate remains biologically active and stable for an extended duration. This novel method holds promise for identifying and addressing abnormal BMP-2 signal transduction in diseases such as osteoporosis and osteosarcoma (OS) [129]. In addition, previous studies have used internalized antibodies to create internalized-QD (iQD) antibody conjugates, and studied the internalization and toxicity characteristics of these conjugates using various visual parameters, indicating that iQD can be effectively internalized into cells [130]. Further research on animal models of rats and rabbits subsequently showed that iQD labeled MSCs and internalized antibodies correspond to the heat shock protein-70 family of stress-induced death proteins. Cells can undergo differentiation of normal bone and chondrocytes in vivo and in vitro, making iQD efficient, gene non-invasive, functionally inert, sensitive, and long-term imaging tools for labeling MSCs in vivo and in vitro [131]. This type of iQD has been used to track MSCs in rabbit models with cartilage defects, implanting three-dimensional cartilage aggregates to the defect site. In fluoroscopy evaluation, iQD tracking is mainly in the bone marrow matrix, detecting signals in the subchondral bone layer. At the same time, iQD provides a stable fluorescent signal during post-graft cartilage repair. It neither inhibits the regeneration of bone and cartilage defects, but also promotes the regeneration of bone and cartilage defects, providing new possibilities for the development of BTE [132]. Conjugated QDs are employed for single-cell labeling within the bone marrow by employing a coating of tetrazine functional groups and utilizing bioorthogonal chemistry with polyimidazole ligands. Through this method, QD antibodies can efficiently spread throughout the bone marrow, effectively labeling individual cells, including rare hematopoietic stem cells and progenitor cell populations [133]. This in vivo cytology technique holds potential for a broad spectrum of structural and functional imaging applications, facilitating the study of cellular interactions with their surrounding environment in both healthy and diseased tissues.

Various QDs have been prepared based on their fluorescence properties, but the most popular research is on CQDs. The primary application of CQDs lies in the realm of biological imaging due to their biocompatibility, low toxicity, and fluorescence properties within living systems [134]. CQDs synthesized from carbon nanocarriers exhibit high affinity and specificity for binding to calcified bone in vivo. Importantly, this binding is non-toxic. This binding characteristic may be attributed to the surface chemical properties of CQDs formulations, making them promising as highly bone-specific biological imaging agents [135]. A study has also shown that QDs can label cells in hydrogel scaffolds and induce bone differentiation (Fig. 6D) [126]. The novel bioactive CQDs prepared from adenosine and aspirin enable long-term fluorescence tracking of human bone marrow MSCs. In addition, studies have shown that CQDs can promote osteogenic transcription and enhance matrix mineralization, even in the absence of any external osteogenic factors, while guiding hmsc osteogenic differentiation [136]. Surface modification and doping of CQDs epresent popular research directions. The high abundance of monodentate phosphonates allows for strong binding to the main bone mineral component, hydroxyapatite. This property enables the preparation of luminescent probes for bone imaging using spherical CQDs bound with monophosphate esters. The characteristics of CQD monophosphate have been validated in an in vitro model of bovine cortical bone, demonstrating the feasibility of imaging microcracks and calcium-rich regions (Fig. 6F) [127]. A sulfur rich S-CQDs with good affinity for bone cells was prepared using a one pot hydrothermal method. As the excitation wavelength changes, S-CQDs can exhibit different tunable colors, providing a rich variety of fluorescence color choices for cell imaging. S-CQDs, combined with their low cytotoxicity and sulfur rich functional groups, can serve as fluorescent probes to detect and differentiate bone related lesion cells [18]. A novel type of Mg-doped CQDs synthesized from metal gluconate salts has emerged. These CQDs can function as cell markers and nanocarriers for Mg^2+^ to enter cells. Once inside, the biologically essential metal ions stimulate osteoblast differentiation by enhancing ALP activity and upregulating related mRNA expression. This holds promising potential for the treatment of osteoporosis [137]. As a special type of CQDs, GQDs have the same low toxicity and good biocompatibility, and N-GQDs are synthesized by doping nitrogen. The nanoscale size and excellent dispersibility of N-GQDs contribute to their enhanced cell permeability. Furthermore, their inherent photoluminescence properties enable the labeling of cells with high uniformity and photostability, eliminating the need for chemical dyes. Importantly, when rBMSCs are cultured with N-GQDs, the expression of key markers such as ALP, extracellular matrix proteins, OPN, and cyanate is upregulated. These findings suggest that N-GQDs possess the capability to promote the osteogenic differentiation of rBMSCs [138]. However, in the absence of nitrogen doped precursors, Alendronate (Alen) can be used to enhance bone affinity through a simple hydrothermal method. Alen CQD exhibits strong binding activity towards calcium deficient hydroxyapatite scaffolds, rat femurs, and live zebrafish. Due to the presence of bisphosphonate groups on the surface of CQD, Alen CQD exhibits excellent bone targeted delivery. This is the first direct bone targeted delivery in vitro, in vitro, and in vivo, supporting the potential application of fluorescent CQDs in the treatment of various skeletal diseases(Fig. 6B) [125].

QDs, an inorganic fluorescent substance, can overcome the shortcomings of traditional organic fluorescent groups, resist light loss, enzymatic degradation in living cells, and a series of chemical damage. Its unique spectral characteristics, excellent photostability, and good resistance to metabolic degradation make it an ideal fluorescent probe for long-term in vivo, multi-target, highly specific, and highly sensitive imaging of cells. Although QDs have achieved many successes in the development of fluorescence imaging and diagnostic probes, there are still some limitations, such as their relatively low affinity for proteins and sensitivity to the existing environment. These limitations may be alleviated through surface coatings and biological coupling methods, but some modifications can reduce the stability and quantum yield of QDs. This requires more research to balance the two, so that QDs can become a standard tool in the field of imaging tracing faster.

### 3.3 Drug loading and delivery in BTE

In recent years, nanoparticles have been used in drug delivery research. QDs exhibit enormous potential among different types of nanoparticles due to their unique properties. The size of QDs plays a crucial role in determining their delivery as drug carriers. The optimal size is crucial for the targeted transfer of QDs, and the size of excellent charge carriers is not necessarily smaller. Related studies have shown that larger QDs may be absorbed by the reticuloendothelial system, while smaller QDs may be cleared through renal filtration. Therefore, researchers screened QDs as drug carriers between 5 and 20 nm. Based on the excellent osteogenic effect of QDs, they are often used as drug delivery for bone diseases.

**Fig. 7:**
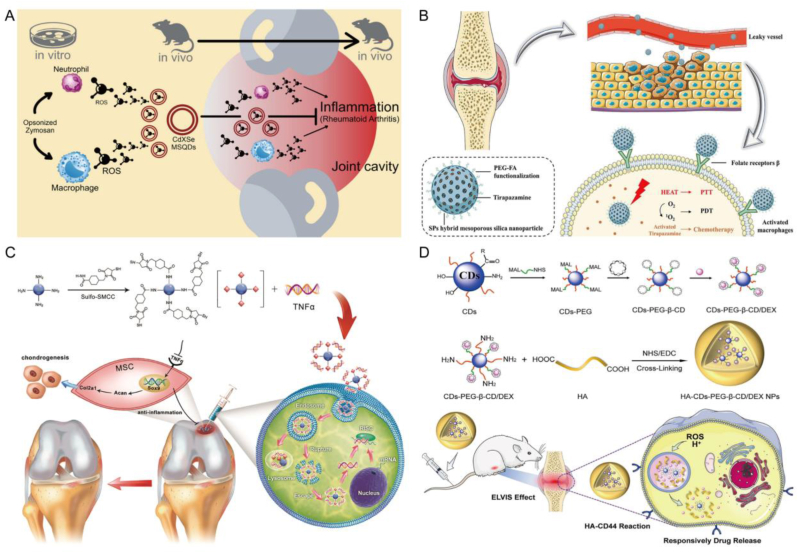
**QDs for drug loading and delivery in BTE.** A) CdSe MSQDs exhibit a mitigating effect on ROS production in neutrophils and macrophages in response to opsonized zymosan stimulation. Reproduced and adapted with permission [140]. Copyright 2022, Future Medicine Ltd. B) Nanoparticles prepared based on QDs can store TPZ and are modified with PEGyl-folate to target activated macrophages in RA. Reproduced and adapted with permission [146]. Copyright 2021, Elsevier. C) Construct bioconjugated carbon points for the delivery of siTnfα, which can enhance chondrogenesis of MSCs by inhibiting inflammation. Reproduced and adapted with permission [147]. Copyright 2019, John Wiley and Sons Ltd. D) HCPC NPs based on CD synthesis enhances the treatment of RA through passive targeting, M1 macrophage targeting, and reactive drug release. Reproduced and adapted with permission [148]. Copyright 2023, Elsevier Ltd.

Among the various types of QDs, those based on cadmium are renowned for their exceptional brightness and resistance to photobleaching during emission [139]. Therefore, this type of QDs can be used to develop traceable nanoscale drug delivery systems. Additionally, certain studies have revealed that CdSe QDs possess the capability to scavenge ROS, making them a promising candidate as an antioxidant molecule with potential applications in the treatment of rheumatoid arthritis (RA) (Fig. 7A) [140]. Some studies have used CdTe QDs coated with thioglycolic acid as nanocarriers for quercetin to neutralize various free radicals produced at the inflammatory site, inhibit the production of COX-2 enzyme, and achieve anti arthritis activity [141]. Experiments have shown that even at lower drug concentrations, nanocarriers have certain anti arthritis effects, demonstrating the potential to enhance anti arthritis drugs in the complications of RA. Myelodysplastic syndrome (MDS) encompasses a highly diverse range of myeloid disorders distinguished by a reduction in peripheral blood cells and an elevated propensity for progression to acute myeloid leukemia (AML). Daunorubicin (DNR) stands as a critical pharmaceutical agent in treating both MDS and AML. Nonetheless, its usage is constrained by adverse effects such as cardiotoxicity and bone marrow suppression, significantly curtailing its clinical utility. In this regard, CdTe QD loaded with DNR and anti-CD123 monoclonal antibodies, an interleukin-3 receptor (IL-3R) chain, binds to DNR-CdTe-CD123 and successfully constructs a new drug delivery system, providing a new platform for MDS treatment [142]. In a separate investigation, CdSe/ZnS QDs were employed to modify fluorescent gelatin nanospheres (GNs) for potential treatment of OS. These modified QDs-GNs were integrated with anti-human immunoglobulin G Fab (anti IgG Fab) to create targeted nanosystems. The size of GNs particles attached to anti IgG Fab was measured at approximately 480 ± 50 nm, and the resultant hybrid nanocarrier, along with anti IgG Fab, exhibited significant internalization by OS cells. The enhanced cell interface of QDs GNs against IgG Fab is associated with the presence of Fab fragments, indicating their suitability for targeted drug delivery systems [143]. It is widely recognized that the toxicity of cadmium-based materials has been a significant concern affecting their in vivo applications. Cadmium-based QDs, including CdSe QDs or CdTe QDs, can pose substantial toxicity risks attributed to ion leakage and exposure to cadmium content [144,145]. Meanwhile, some studies have found that CdQDs have a certain inhibitory effect on the osteogenesis of hBMSCs, so more research is needed to make various modifications to determine their effectiveness and safety.

Of course, other types of QDs can also serve as carriers for drug delivery. Alendronate sodium is a bisphosphonate drug used clinically to prevent osteolysis and alleviate pain. It has been demonstrated that silver sulfide QDs can be surface-bound for targeted delivery to bone, aiming to inhibit osteolysis. Additionally, a conventional anti-tumor drug, doxorubicin (DOX), has been encapsulated within a hydrophobic layer surrounding Ag_2_S QDs for the purpose of tumor chemotherapy. The straightforward surface engineering treatment of Ag_2_S QDs ensures prolonged circulation time of the nanomedicine, thereby circumventing uptake by the mononuclear phagocytosis system and enhancing opportunities for targeted regional delivery. The high affinity of Alen ligands for bone tissue enables nanomedicines to pass through the blood bone marrow barrier and achieve better therapeutic effects, while inhibiting bone resorption by regulating bone homeostasis. When the acidic tumor microenvironment is triggered, DOX is released on demand at the tumor site, specifically killing cancer cells. The rapid and specific deposition of this type of Ag_2_S QDs nanosystem in bone tissue is the first demonstration of multifunctional Ag_2_S QDs nanodrugs in situ bone tumor models using chemotherapy and bone lysis inhibition [149]. In an innovative study, Cetroxonium bromide was employed for the first time as a stabilizer to ensure the stability of semiconductor polymer QDs (SPs) in vitro. Additionally, it served as a template for the successful preparation of SP hybrid mesoporous silica nanoparticles (SMs). This novel design notably enhanced the long-term stability of SPs in vivo. The mesoporous properties of these nanoparticles provide an ideal storage space for hypoxia-activated prodrug tilaprazine (TPZ). In order to achieve precise targeting of activated macrophages in RA, the researchers further modified PEG-folate (PEG-FA) onto the SMs surface to form SMPFs, significantly enhancing the affinity of nanoparticles to activated macrophages in RA lesions. During the treatment, the local anoxic environment, due to the consumption of molecular oxygen, triggers the cytotoxic activity of TPZ, thus effectively eliminating these inflammatory cells. This mechanism not only directly inhibits the activity of activated macrophages, but also slows down the pathological process of RA, demonstrating the great potential of this new nanomedical drug in the treatment of RA (Fig. 7B) [146].

The core structures of carbon based QDs are different and can be divided into CQDs and GQDs, respectively. The fluorescence, water solubility, biocompatibility, low toxicity, small size, and easy modification have garnered significant interest in the realm of drug delivery [150]. A study reported an integrated GQDs nanosystem based on fluorinated (F-GQDs), whose aromatic conjugated structure and excellent water solubility provide possibilities for many anti-inflammatory drugs, which are absorbed through π - π stacking, hydrogen bonding, and hydrophobic interactions. F-GQDs loaded with drugs have good biocompatibility and the ability to track cellular uptake and drug release, and upregulate the cartilage synthesis metabolism gene Col-2α and aggregation proteins provide protective anti-inflammatory potential against hydrogen peroxide induced degradation of chondrocytes. However, the system can not only load anti-inflammatory drugs, but its ultra small size and nearly spherical rolling effect demonstrate good lubrication performance, achieving visual monitoring of drug release and providing new ideas for the treatment of osteoarthritis [151]. In addition, the researchers used GQDs as a drug carrier targeting BMP-2 for bone therapy. As a drug carrier, polysaccharide GQDs gels have 3D network structure, large specific surface area and excellent drug delivery performance. The experiment proved that the BMP-2 bone targeting drug carrier system has been improved, and the drug carrier system has high bone targeting selectivity, and is a good drug carrier with bone targeting effect.

In the current trend, the administration process of CQDs has received more comprehensive research. So far, it has shown that the synthesis of black carbon powder by CQDs is non-toxic and has a unique ability to bind to calcified bone tissue. They chemically modify CQDs, such as surface modification with ethylenediamine or glutamic acid [135], without interfering with in vivo affinity and specificity, providing principles that may be used for bone specific drug delivery. At present, the treatment of osteoporosis mainly relies on preventing further bone erosion [152]. CQDs can bind to bone growth areas without interfering with normal renewal, repair, and regeneration in the body [153], which also provides the possibility of drugs for treating diseases such as osteoporosis. In addition, the green formulation of CQDs has brought new ideas for their drug delivery. Some studies have synthesized CQDs from biological waste precursors, which combine with hydroxyapatite (HAP) to form nanocomposite CQD-HAP and serve as a drug carrier for acetaminophen treatment of osteoarthritis. The experimental findings suggest that the incorporation of CQDs with HAP can enhance drug loading capacity, rendering it an effective approach for drug delivery applications aimed at improving treatment efficacy [154]. However, a single CQDs has limited properties, and a study has been conducted to construct multifunctional drug nanocarriers by directly conjugating two different CQDs. In the experiment, the bone-targeting CQDs and the gel-like CQDs that can penetrate the blood-brain barrier were conjugated through amidation reaction, and a new type of CQDs inheriting the two characteristics was formed. In addition, the study found that the effect of improving the loading capacity of drugs can be achieved by adjusting the conjugation ratio, and this Lego design opens the way for new and efficient drug delivery systems [155].

The research on CQDs is not limited to demonstrating their drug loading and delivery capabilities. Many studies have focused on specific bone diseases as CQDs loaded related therapeutic drugs to achieve the goal of treating diseases. RA is an autoimmune disorder characterized by inflammation that progressively damages the joints, resulting in joint inflammation, polyarthritis, and the erosion and deformation of bones and cartilage [156]. In this regard, some studies have combined musSK monoterpenes with CQDs to achieve anti rheumatic ability. Following successful preparation, experiments have revealed that CQDs possess the capability to deliver the anti-arthritis agent thymol. The bioactive compounds of thymol exhibit potent anti-arthritis activity against targets associated with RA. Moreover, in vivo studies have demonstrated that CQDs can mitigate bone destruction, joint disorders, and swelling in arthritis-induced Wistar rats [157]. However, for the treatment of RA, a sufficient dose of dexmedetomidine is required at the inflamed site to produce anti-inflammatory effects, but the low loading efficiency of CQDs may limit their application in DEX administration and affect drug activity. Thus, in a particular study, β-cyclodextrin (β-CD) was grafted onto the surface of CQDs through functional groups, resulting in the formation of β-CQDs. This modification endowed the β-CQDs with a hydrophilic shell and a hollow cylindrical hydrophobic inner cavity, facilitating easy drug loading. Leveraging hyaluronic acid, the research team successfully engineered HCPC/DEX NPs, a delivery platform targeting M1 macrophages and responsive to environmental cues. These nanoparticles efficiently transported DEX to the inflammatory site within RA joints. By inhibiting the inflammatory response of M1 macrophages, they play a crucial role in RA treatment while mitigating the adverse reactions associated with DEX administration (Fig. 7D) [148].

Developing an effective delivery system to promote the penetration of bioactive factors or drugs through the dense surface structure of cartilage to the deep and repair cartilage damage is a key and unresolved issue. Researchers focused on the synthesis of *m*-CQDs utilizing heavy morphine diamine and subsequently devised a novel multifunctional delivery system tailored for chondrocytes and cartilage. This delivery system exhibits rapid transfection capability (within 30 min), high transfection efficiency (over 90 %), exceptional biocompatibility (maintaining 92 % cell activity), and stable chondrocyte photoluminescence. These attributes enable efficient penetration of bioactive factors through the dense structures on the cartilage surface into deeper cartilage areas, thereby significantly enhancing their therapeutic efficacy [158]. Although this is a promising strategy, the treatment of cartilage defects based on MSCs may sometimes be accompanied by chronic inflammation in the reconstructed state, which may also hinder cartilage regeneration. Inflammatory factor tumor necrosis factor α (TNF-α) has played an important role. Some studies have used CQDs and sulfosuccinimide-4- (N-maleimide methyl) cyclohexane-1-carboxylate (graft SMC) to biologically replicate CD-SMC for the transfer of silenced TNF in bone marrow MSCs. The findings revealed that CD-SMC siTnF-α effectively interfered with TNF-α, resulting in a significant promotion of chondrogenesis in bone marrow mesenchymal stem cells. This effect was characterized by the upregulation of cartilage-specific markers and accelerated cartilage regeneration. These results highlight the potential of gene therapy for applications in cartilage tissue engineering (Fig. 7C) [147].

QDs possess small size and versatile surface chemical properties, enabling them to readily bind with various nanoparticle drug delivery carriers without significantly altering their overall properties. Additionally, QDs offer exceptional optical properties, facilitating real-time monitoring of transport and drug release at both cellular and systemic levels. At present, the biggest controversy over QDs is their potential long-term toxicity, but studies have shown that their safety and biocompatibility in vivo can be ensured by using green and safe QDs or replacing their core with non-toxic organic drug carriers. Although QDs have made some progress in their research as delivery systems, their application is still in its early stages. The limitation of its development is probably due to the current lack of understanding of QDs nanostructures, which can interfere with their binding as carriers and some drugs. In addition, research data on how QDs release drugs from the core to the target, and how factors such as size affect their uptake and transport to cells is still insufficient, which slows down the progress of effective treatment of QDs and limits their practicality in the field of delivery. We look forward to more research investment, so that QDs can become a powerful tool for drug loading and delivery in BTE as soon as possible.

### 3.4 Antibacterial properties in BTE

Bacterial infection and inadequate bone integration are the two major problems that plague individuals in repairing bone defects. Infectious bone defects arise from bacterial adhesion and biofilm formation on the implant surface. For instance, exposure to Gram-positive Staphylococcus aureus can significantly impair local tissue and bone regeneration, rendering the defect challenging to heal [159]. Current conventional treatments, such as necrotic bone removal, antibiotic administration, and bone defect reconstruction, are time-consuming and often fail to achieve satisfactory effectiveness. There are relevant literature said that various materials have excellent antibacterial ability or osteogenic activity [160,161], however, a single function of artificial implant material is not enough to repair the infected bone defect. Therefore, it is very necessary to develop materials with both antibacterial and osteogenic functions. Based on the excellent osteogenic ability of QDs, the researchers studied them in depth and found that because of their ultra-low size and high surface activity, QDs can quickly attach and accumulate on bacterial cells, effectively destroy the bacterial membrane through large contact stress, and eventually lead to bacterial death. In particular, carbon-containing QDs, including GQDs and CQDs, exhibit bacteriostatic and bactericidal activity through photodynamic (PD) effects against antibiotic-resistant bacteria at a certain wavelength of light [162]. In addition, QDs can also produce ROS to destroy cells, and combine with accounting substances to inhibit cell proliferation [21]. Given these advantages, its application in BTE scaffolds is anticipated to facilitate bone repair by mitigating early inflammation and excessive immune responses triggered by inhibitory material implantation. This, in turn, would establish an optimal microenvironment for bone regeneration.

In a study, GQDs were integrated into TiO_2_ nanorods via hydrothermal treatment to yield G-TiO_2_ NR. The modified TiO_2_ nanorods demonstrated the capability to disrupt the integrity of bacterial cell membranes, exhibiting outstanding antibacterial efficacy against S. mutans, with up to 97.8 % antibacterial ability observed in vitro. Additionally, in vivo antibacterial testing and evaluation of osteogenic activity were conducted. The findings revealed that G-TiO_2_ NR exhibited robust in vivo antibacterial activity and effectively stimulated new bone formation even in the presence of bacterial infection [163]. In another study, CQDs were synthesized from coffee grounds using a microwave-assisted hydrothermal method. Subsequently, these CQDs were incorporated into polylactic acid (PLA) porous membranes via direct electrospinning. The promise of these modified PLA membranes in guided bone and tissue regeneration was evaluated by assessing their biomimetic mineralization and antibacterial properties. The results showed that after 24 h of composite membrane culture with QD modification, the number of S. aureus and Escherichia coli decreased by 94 %, and in a certain range, the higher the QD, the antibacterial effect was better, and the real-time and broad-spectrum antibacterial properties, which may be due to the synergistic effect of membrane stress and oxidative stress conferred by QD [164].

**Fig. 8:**
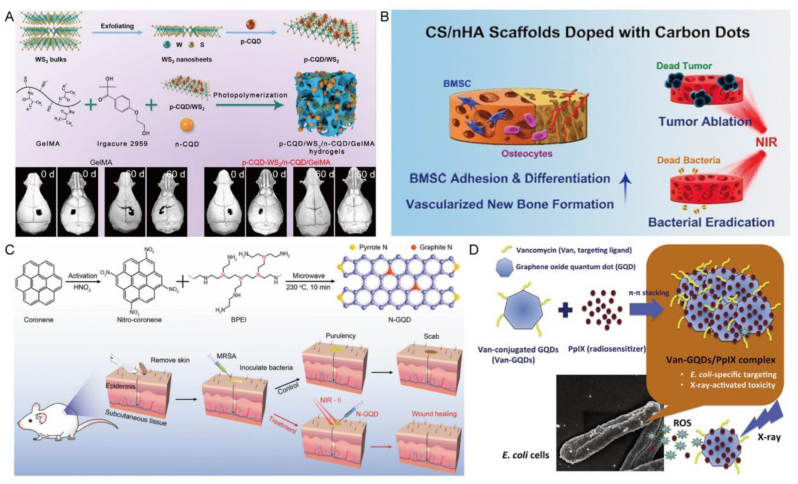
**QDs for antibacterial agent in BTE.** A) The hydrogel scaffolds synthesized based on CQDs and their antibacterial properties were used for the regeneration of bone defects infected by multi-drug resistant bacteria. Reproduced and adapted with permission [165]. Copyright 2021, Elsevier Ltd. B) The composite scaffold based on CD synthesis promotes bone formation and anti-tumor, and also has obvious antibacterial properties against Staphylococcus aureus and Escherichia coli collected in clinic. Reproduced and adapted with permission [166]. Copyright 2018, American Chemical Society. C) The preparation process of N-GQDs and its excellent antibacterial and antibiofilm activity against MDR bacteria present under laser irradiation. Reproduced and adapted with permission [167]. Copyright 2022, Royal Society of Chemistry. D) GQDs coupled with vancomycin were assembled with protoporphyrin IX to construct a complex that can play a bactericidal role against E. coli. Reproduced and adapted with permission [168]. Copyright 2017, Elsevier BV.

Considering that the emergence of multidrug resistant (MDR) bacteria reduces the bone inducibility of stent materials, a study has reported for the first time to avoid the aggregation of positive and negatively charged components by changing the surface charge of CQDs. The research team was previously aware that negatively charged CQDs had a considerable potential to enhance bone regeneration [118]. Experimental data reveals that positively charged CQDs (*p*-CQDs) exhibit a broad spectrum of antibacterial activity against non-multi-drug resistant bacteria, such as Escherichia coli and Staphylococcus aureus, owing to strong electrostatic and hydrophobic interactions with the negatively charged bacterial membrane. Moreover, *p*-CQDs also demonstrate some level of antibacterial efficacy against multi-drug resistant bacteria (MRSA). This antibacterial activity has been further validated through in vivo experiments, affirming the promising potential of *p*-CQDs as effective antibacterial agents. Combined with its advantages of promoting bone regeneration, it has great potential for antibacterial and treatment of osteogenic active bone defects in infected patients (Fig. 8A) [165].

Numerous non-antibiotic strategies, including photocatalysis and photodynamic therapy, have been proposed as alternative approaches to inhibit and eradicate bacteria. Among which light-triggered antimicrobial therapy has been shown to be a safe and effective treatment for bacterial infections. Geng et al. has for the first time prepared N-GQDs demonstrate exceptional antimicrobial efficacy and antibiofilm properties against MRSA (such as methicillin-resistant Staphylococcus aureus), particularly under specific laser conditions, owing to their high performance in IR-II photothermal conversion for antimicrobial therapy. In vivo experiments have shown that N-GQDs combined with IR-II laser irradiation can significantly accelerate the healing of infected wounds. For the epidermal trauma caused by the implantation of scaffolds into the bone, we believe that this GQDs is expected to be a new antibacterial agent to prevent bacterial infection during healing (Fig. 8C) [167]. In a study, the antimicrobial efficacy of CDs was tested by regulating NIR light. The study compared the antibacterial effects of CS/nHA/CD and CS/nHA stents, and concluded that the incorporation of CD resulted in a significant increase in antibacterial activity upon NIR irradiation of the stents, particularly against pathogenic strains of S. aureus and E. coli. This enhancement suggests the potential to effectively eliminate clinically relevant bacterial infections (Fig. 8B) [166]. In another study, CQDs with excellent biocompatibility were integrated into TiO_2_ nanorods to enhance the photocatalytic and photothermal properties of titanium implants under visible light (VL) and NIR irradiation. As a result of the synergistic effects of hyperthermia, ROS, and the nanorod structures, C–TiO_2_ NR exhibited outstanding antibacterial effects both in vitro and in vivo when co-irradiated with 660 nm VL and 808 nm NIR light. Additionally, C–TiO_2_ NR enhances the adhesion and proliferation of BMSCs. Consequently, this photoassisted antimicrobial therapy is deemed a safe and efficacious approach for preventing bacterial infections in clinical applications [169].

Nevertheless, these methods still exhibit certain drawbacks, including insufficient bacterial specificity and the limited penetration depth of UV and NIR light. To address these limitations, researchers have explored a bacteria-specific antimicrobial technique utilizing low-dose X-rays. The GQDs were conjugated with vancomycin (Van) and combined with protoporphyrin IX (PpIX) to create a novel Van-GQDs/PpIX complex. Through this formulation, GQDs facilitated the bacterial targeting of both Van and PpIX, resulting in an elevated PpIX/Van ratio within bacteria. Consequently, systemic toxicity was reduced, and targeted delivery of PpIX was enhanced. Furthermore, the Van-GQDs/PpIX complexes were found to disrupt the bacterial cell wall, ultimately resulting in the demise of E. coli. Short-term resistance tests conducted revealed that the surviving bacteria did not develop resistance to the X-ray-activated Van-GQDs/PpIX complexes. Therefore, this vector is a promising alternative to antibiotics, advantageous for the treatment of severe bacterial infections like osteomyelitis that occur in deep tissues (Fig. 8D) [168].

Besides the two classes of QDs, there are other QDs that show excellent antimicrobial capability and are applied in BTE. Zn serves as a crucial component of ALP and acts as a pivotal mediator in bone matrix mineralization, thereby playing a significant role in bone formation. Meanwhile, the zinc ions have an antibacterial effect, which is beneficial to inhibit the infection. In comparison to other types of QDs, ZnO QDs are relatively less cytotoxic to cells and environmentally friendly, making them a widely utilized and effective antibacterial agent. A study employing ZnO QDs modified BGN to create ZnO-BGN nanocomposites has demonstrated enhanced inhibition of both gram-positive Staphylococcus aureus and gram-negative Escherichia coli. Moreover, these nanocomposites have shown the ability to enhance the osteogenic differentiation of hMSCs. Due to their apatite formation capability, distinctive ion release behavior, lack of cytotoxicity, antibacterial properties, and osteogenic potential, synthetic ZnO-BGN nanocomposites are considered promising for bone regeneration applications [91]. A bifunctional acoustic sensitizer G-ZnN_4_-MoS_2_ composed of porphyrin Zn single atom catalyst (G-ZnN_4_) and MoS_2_ QD has been developed. It was found that ^1^O_2_ generated at the O_2_ interface kills MRSA, with an antibacterial efficiency of up to 99.58 %, and G-ZnN_4_-MoS_2_ also promotes osteogenesis through the continuous release of Zn^2+^ from fixed Zn single atoms. Such acoustic sensitizers with excellent sonodynamic antimicrobial efficiency and osteogenic capacity provide a new strategy for treating osteomyelitis by sonamic ions [170].

The main pathogens of bone infection are Gram positive cocci, anaerobic bacteria, and Gram negative anaerobic bacteria. Studies have shown that QDs have certain inhibitory effects on common bacteria in bone infections, but bone infections are influenced by various bacteria and fungi. Currently, there is no literature indicating the mechanism of QDs in antifungal aspects. At the same time, research on the antibacterial mechanism of QDs is still incomplete, and there is insufficient understanding of their selectivity towards microbial communities. In addition, bacteria may develop resistance to QDs, and even though there are studies addressing resistance issues, more efforts are still needed to support QDs becoming standardized and regulated antibacterial materials as soon as possible.

### 3.5 Phototherapy in BTE

In recent years, phototherapy has become increasingly utilized in the treatment of bone cancer. This therapeutic approach, encompassing both PTT and PDT, offers an appealing non-invasive method for remotely activating medications to treat a range of diseases [171]. In the process of phototherapy, phototherapeutic agents are administered to the lesion site and subsequently exposed to a precise wavelength of light. In PDT, the PS is activated, triggering the generation of ROS that lead to irreversible cell damage [172,173]. In the PTT, the photothermal agent absorbs NIR light, resulting in the generation of heat that directly induces cell ablation [174,175]. Researchers have the capability to convert the irradiated light into reactive oxygen species or heat to trigger local apoptosis of tumor cells and eliminate residual tumor cells in the body. Due to their widely adjustable fluorescence properties, excellent photostability and chemical stability, as well as remarkable solubility and biocompatibility, QDs can be used as promising next-generation material drugs to drive phototriggering PDT and PTT.

**Fig. 9:**
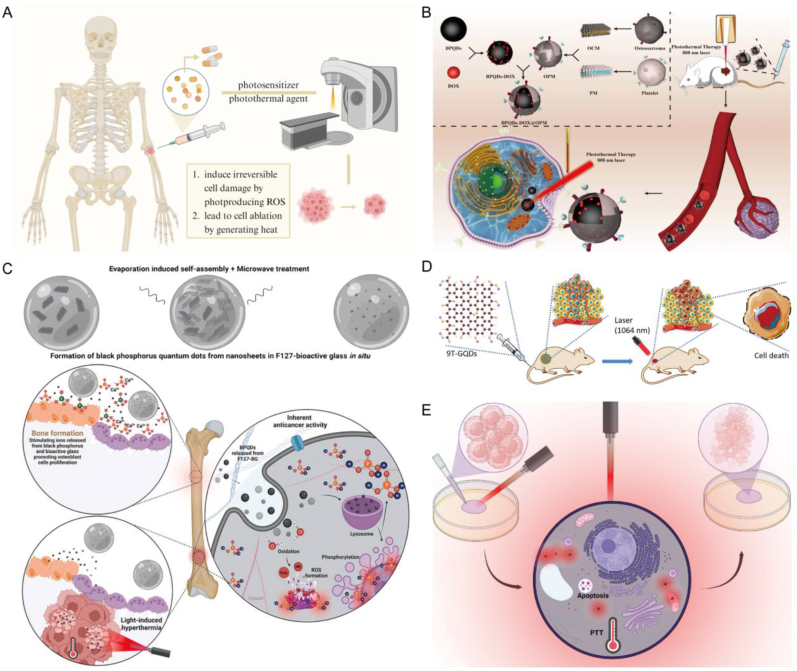
**QDs for phototherapy in BTE.** A) Schematic diagram of the application of QDs to BTE under phototherapy. B) A schematic illustration of the preparation of BPQDs-DOX@OPM system and its combination with PTT enhanced drug therapy for OS. Reproduced and adapted with permission [177]. Copyright 2023, BioMed Central. C) Combined with NIR hyperthermia, a nanocomposite material for the treatment of bone cancer and bone tissue regeneration was constructed based on BPQDs. Reproduced and adapted with permission [179]. Copyright 2024, KeAi Communications Co. D) Imaging-guided PTT of 9T-GQDs irradiated under NIR-II laser. Reproduced and adapted with permission [180]. Copyright 2020, Elsevier BV. E) Schematic diagram of anti-cancer potential induced by F127-BG-BPQDs hyperthermia in vitro. Reproduced and adapted with permission [179]. Copyright 2024, KeAi Communications Co.

Bone tumors represent one of the most prevalent types of tumors, with conventional treatments typically involving surgery, often supplemented by radiotherapy (RT) and chemotherapy. OS stands out as the most common primary malignant bone tumor, however, the use of RT for OS has been limited and is prone to drug resistance in the process. In one study, the scientists developed a novel nanoscale radiosensitizer, 2DG-g-GQD. This sensitizer can specifically locate primary and distal OS in vivo and significantly inhibit tumor growth when used in combination with X-rays (6 Gy). 2DG-g-GQDs can also inhibit the migration and invasiveness of OS cells and induce oxidative stress response. But the single RT strategy still has some shortcomings, for which the researchers turned to light therapy [176]. Xu et al. have devised a clever strategy that combines PTT with chemotherapy, resulting in a novel therapeutic approach. They engineered a platelet-osteosarcoma mixed membrane (OPM) to act as a protective shell, enclosing multiple BPQDs loaded with the anticancer drug DOX. This innovative design enables the drug to be released more rapidly upon exposure to NIR irradiation, thereby extending the circulation time and achieving light-triggered drug release. Both in vitro and in vivo studies have shown that the BPQDs-DOX@OPM system efficiently delivers the therapeutic payload to the tumor site, offering improved circulation longevity and targeted delivery. This dual-modality therapy demonstrates superior antitumor efficacy compared to conventional single-agent chemotherapy, representing a significant advancement in cancer treatment strategies (Fig. 9B) [177]. GQDs can repeatedly form Vapor Nanobubbles under multiple laser irradiation, thereby improving the efficiency of intracellular delivery. This undoubtedly provides a theoretical basis for QDs in phototherapy, and the ability of GQDs to withstand multiple laser exposures without being destroyed means that they can be reused in the phototherapy process, which has potential value in reducing treatment costs and improving treatment efficiency [178].

Previous studies have investigated the potential of graphite-phase carbon nitride (G-CN) QDs in microwave-induced photodynamic therapy (MIPDT). The initial observation revealed that microwave irradiation induces the production of singlet oxygen in G-CN QDs, offering a means for tumor destruction. Various experimental findings indicate that G-CN QDs exhibit minimal cytotoxicity and excellent biocompatibility in physiological conditions. Utilizing G-CN QDs in MIPDT proves effective in killing cancer cells and promoting tumor cell death [181]. In another study, GQDs were treated with phenol under a high magnetic field of 9T, facilitating the adjustment of H_2_O_2_ decomposition. The resulting nanomaterials were designated as 9T-GQDs. These 9T-GQDs exhibited tunable fluorescence properties and achieved a high photothermal conversion efficiency of up to 33.45 %. Both in vitro and in vivo experiments demonstrated that 9T-GQDs could effectively ablate tumor cells and suppress tumor growth when subjected to laser irradiation in the NIR-II region (Fig. 9D) [180].

The construction of Ra-GQDs using GQDs as a vector for radium (Ra) is also a promising approach because GQDs have low toxicity, high biocompatibility and sufficient tumor penetration size. In a particular study, researchers assessed the treatment of bone cancer using GQDs radiolabeled with Ra both in vitro and in vivo. The findings revealed significant effectiveness in reducing cell viability in OS cells (MG63 and SAOS 2), while displaying less impact on normal fibroblasts, indicating preferential targeting. Moreover, the results highlighted a more pronounced effect on MG63 cells compared to SAOS 2 cells, suggesting targeting toward more undifferentiated cells, which are consistent with those observed in vivo. These outcomes underscore the efficacy of Ra-GQDs and offer promising avenues for their utilization in various other diseases [182]. Bigham et al. designed a new type of regenerative nanocomposite material based on QDs, which was composed of bi-tropic nonionic surfactants (Pluronic F127), BG and BP, and tested the inherent anticancer activity of the nanocomposite containing BP on OS cells in vitro, and evaluated the apoptosis pathway (Fig. 9C). The application of near infrared irradiation to induce further inhibition of cell proliferation through hyperthermia has achieved certain anticancer effect (Fig. 9E) [179].

Phototherapy has also been shown to directly regulate the regeneration of bone, cartilage, and muscle by regulating cell behavior. Recently, a study designed a self-assembled double-layer hydrogel with a spatiotemporally-modulated immune microenvironment for osteomyelitis treatment. In this system, the top layer AC_10_A hydrogel (AA) is designed to be loaded with Ag_2_S QDs@DSPE-mPEG_2000_-Ce6/aptamers (AD-Ce6/Apt). AD-Ce6/Apt can effectively target and kill Staphylococcus aureus under laser irradiation. Furthermore, AD-Ce6/Apt has the capability to induce macrophages to undergo M1-type polarization, thereby activating the bone immune system. In the lower layer, AC10ARGD hydrogel (MAR) is infused with BMSCs. Shielded and bolstered by the MAR layer, these stem cells can differentiate into osteoblasts and facilitate bone tissue healing. Simultaneously, BMSCs released from the MAR layer can also foster the formation of an anti-inflammatory microenvironment by modulating the M2-type polarization of macrophages [183]. It follows that the application of QDs in PDT and PTT also favors their role in other aspects.

Although significant progress has been made in the application of QDs as nanocarriers or phototherapeutic agents in PDT and PTT, their relatively low quantum yield and wide emission spectrum remain issues when it comes to the photoluminescence mechanism of QDs, limiting their widespread application. At the same time, there is currently a lack of research on the use of QDs for tissue penetration, which is still limited by visible or NIR light, coupled with low light conversion efficiency, resulting in unsatisfactory therapeutic effects. QDs can be considered in combination with immunotherapy in phototherapy to achieve better therapeutic effects. Finally, more exploration should be devoted to studying the stability of QDs in vivo circulation, achieving their targeting of therapeutic tissue sites and controlled release of phototherapy drugs.

### 3.6 Osteoimmunomodulation in BTE

**Fig. 10:**
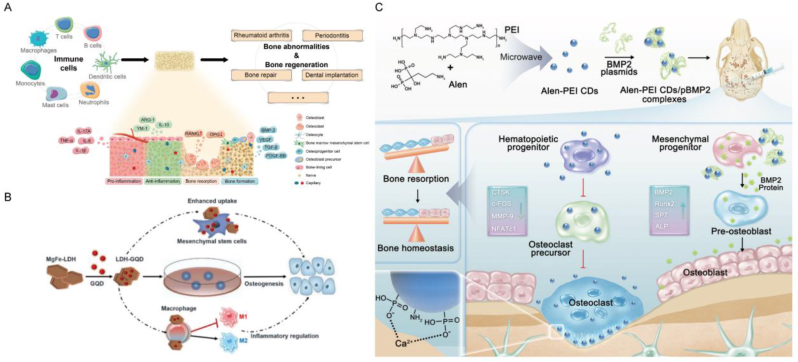
**QDs for osteoimmunomodulation in BTE.** A) Reproduced and adapted with permission [186]. Copyright 2023, MDPI (Basel, Switzerland). B) Schematic diagram of LDH-GQD promoting osteogenic differentiation ofrBMSCs by enhanced cellular uptake and inflammatory regulation. Reproduced and adapted with permission [189]. Copyright 2022, IOP Publishing Ltd. C) Multifunctional fluorescent Alen-PEI CDs can inhibit osteoclasts and significantly reverse the imbalance of bone homeostasis through the bidirectional mechanism of bone immunity. Reproduced and adapted with permission [190]. Copyright 2022, Wiley-Blackwell.

The immune system and the skeletal system have a close relationship, evidenced by the sharing of numerous cytokines, receptors, and signaling molecules between them [184], which has led researchers to rethink the functions of bone and the immune system, thus proposing the concept of bone immunity. The establishment of the relationship between T cells and osteoclasts marks the beginning of bone immunology. The RANKL/RANK/OPG signaling pathway is an important link between the bone system and the immune system, and various cytokines and receptors participate in bone metabolism by regulating the functions of osteoclasts and osteoblasts (Fig. 10A) [185,186]. As one of the earliest infiltrating cell types in bone healing hematoma, macrophages play an indispensable role throughout the healing process. Initially, recruited macrophages are polarized towards the pro-inflammatory M1 phenotype, which facilitates osteogenesis. Subsequently, they transition gradually towards the anti-inflammatory M2 phenotype, thereby promoting the recovery of bone tissue [187,188]. The ideal scaffold material in the field of bone regeneration should satisfy the timely switch from early inflammation of immune cells to late anti-inflammatory, especially the M1 to M2 phenotype switch in the macrophage population. Bone regeneration requires immune regulation to generate an ideal microenvironment for subsequent bone formation, and the properties of QDs, which can be coupled with related cytokines, therapeutic drugs and bioactive ions that regulate the immune system, received a certain amount of attention.

Dex CQDs (DCDs) were prepared by Wan et al. in a hydrothermal method, and research has shown that DCDs have good osteogenic potential under normal or inflammatory conditions. Subsequently, the experiment explored the bone immunomodulative function of DCDs, and the results showed that the DCDs group had the lowest levels of TNF-α and IL-1β secretion, and the highest expressions of OPN, Runx2 and Col 1. By stimulating the transformation of macrophages from M1 type to M2 type, the bone immune microenvironment could be induced and the bone tissue regeneration could be promoted [191]. Wang et al. prepared GQDs of layered double hydroxide (LDH) nanoparticles to study their role in bone immunity. By stimulating macrophages with myelin protein (MBP) to simulate the inflammatory environment, LDH-GQD was found to significantly inhibit the expression of MBP-induced M1-type macrophage marker TNF-α and up-regulate the expression of M2-type macrophage marker IL10. This suggests that LDH-GQD has anti-inflammatory effects and helps to regulate the immune microenvironment, which is conducive to bone regeneration (Fig. 10B) [189]. At the same time, M2 macrophages are able to release factors that promote angiogenesis, such as VEGF, thereby supporting new blood vessel formation and bone tissue repair. In two studies, He et al. first pointed out that BPQDs had limited direct angiogenesis of endothelial cells, but they could indirectly promote vascularization and bone regeneration by regulating the polarization of macrophages. Later, through the interaction of BPQDs with adipose-derived MSCs (ADSCs), it was indicated that BPQDS can significantly improve the osteogenic differentiation ability and regulate the bone immune microenvironment. BPQDs can enhance osteogenic differentiation of ADSCs, which is regulated by Wnt/β-catenin and BMP2/SMAD5/Runx2 signaling pathways. In addition, BPQDs pretreated ADSCs can promote the osteogenic effect of BMSCs through the paracrine pathway and promote the transformation of macrophages from the pro-inflammatory M1 type to the pro-healing M2 type in the periodontitis microenvironment, which helps to accelerate angiogenesis and bone regeneration processes [192].

T cells affect bone cells, especially osteoclasts, and are closely related to bone biology [193]. Cytokines that regulate immune function, such as TNF-α, IL1, IL-11, and interferon-γ (IFN) -γ, promote immune regulation by inhibiting the proliferation and recruitment of various pro-inflammatory immune cells [194]. Rafieerad et al. has developed an 0D Ti_3_C_2_-based MXene QDs (MQDs) for enhanced tissue repair after injury. MQDs have shown immunomodulatory ability to reduce the activation of specific T cells and promote the expansion of regulatory T cells, which helps to reduce inflammation and promote tissue healing [195].

By affecting osteoclast differentiation, bone immune regulation can also be affected. Chen et al. study showed that the N-CQDs derived from chitosan have good antioxidant properties. By increasing the expression of key antioxidant enzymes and inhibiting the expression of pro oxidants, they can inhibit RANKL induced intracellular ROS production, block MAPK and NF-κ The activation of the B signal cascade inhibits osteoclastogenesis and bone resorption. Such CQDs can reduce lipopolysaccharide induced skull destruction and breast cancer induced tibial bone loss in vivo, and treat osteoclast mediated osteolytic disease at the level of bone immune regulation [196]. Li et al. prepared for the first time a carbon dot PCDs derived from acid apples to reduce bone resorption induced by ultra-high molecular weight polyethylene wear particles after artificial joint replacement surgery. The experimental results indicate that PCDs can avoid ROS mediated osteoclast generation and reduce some pro-inflammatory cytokines, such as TNF-α, IL-1 β, IL-6 and IL-8, expected to treat symptomatic osteolysis and osteoclast-mediated diseases from the perspective of immune regulation [197]. Zhang et al. have successfully developed a multifunctional fluorescent Alen-polyvinylimide CD, which not only effectively promotes osteoblast differentiation and bone regeneration, but also directly inhibits osteoclast activity, thereby significantly reversing the bone resorption process through a bidirectional mechanism in the case of bone homeostasis imbalance. The bone-targeting properties and anti-bone resorption of this nanomedical drug provide a new idea in regulating bone immune response and promoting bone tissue repair (Fig. 10C) [190].

As a nanoparticle, QDs inevitably has some drawbacks, such as biocompatibility, immunogenicity and toxicity, which will limit its development and clinical application. Moreover, there are still few researches on QDs in the field of bone immune regulation, and its mechanism of action lacks a comprehensive explanation, so we cannot ensure the accurate targeting and therapeutic efficiency of QDs in vivo. How to ensure the penetration of QDs to other organs remains a challenge for future research. In general, the development direction of QDs in BTE can be toward surface modification and microenvironment targeting to achieve accurate environmental response in the treatment of diseases.

## 4 Further applications of AI-assisted QDs in bone organoids

When it comes to BTE, it is inevitable to mention the current organoid treatment strategies. Organoid are a 3D constructs derived from stem cells that simulate the structure and function of natural organs, used to study the development and behavior of different organs, as well as drug screening and disease modeling [198]. At present, the construction and application evaluation of bone organoids [[199], [200], [201]] is a hot topic in BTE. Bone organoids can overcome the complex heterogeneity of cell growth and differentiation in the body, providing a physiological environment that improves our understanding of bone development, regulation, and disease mechanisms, ultimately leading to better treatment and outcomes for patients [202]. In view of previous studies used renal organoids to evaluate the nephrotoxicity of QDs [203], we speculated that bone organoids also can be used to evaluate the toxicity of QDs in BTE, deepening the study of the molecular mechanisms of QDs in vivo, in order to find green and safe QDs. In turn, QDs can act as biomarkers and imaging to track the behavior of cells and biomolecules to gain insight into bone tissue growth, repair, and disease processes. In addition, QDs can also be used as a drug delivery system to improve the targeting and efficiency of therapeutic drugs and provide a new therapeutic strategy for the repair of bone organoids. However, the traditional research methods have some problems, such as low efficiency, poor accuracy and insufficient optimization. With the rapid development of artificial intelligence (AI) algorithms such as GPT, AI is recognized as having a key role to play.

**Fig. 11:**
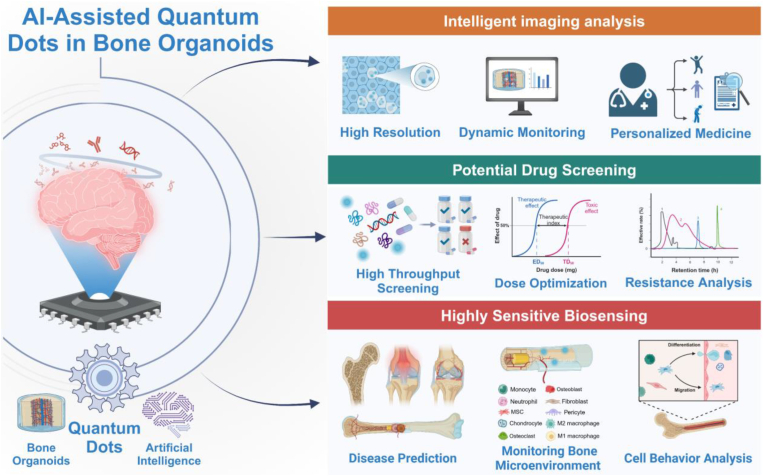
**Further application of AI-assisted QDs in the study of bone organoids.** AI-assisted quantum dots in bone organoid research can promote in-depth understanding of bone tissue development and disease mechanisms through precise imaging, drug analysis and screening, and high-sensitivity sensing, providing a powerful tool for personalized medicine and the development of new therapies.

In the study of QDs and bone organoids, AI can contribute from intelligent imaging analysis, potential drug screening, and highly sensitive biosensing(Fig. 11). (1) QDs has unique optical properties and can be used for biological imaging. AI-assisted QDs combined with bone organoids can provide high-resolution bioimaging to deeply observe cell and tissue structures, achieve dynamic monitoring of bone growth and repair processes, and customize treatment plans according to patients' specific conditions through personalized medical strategies, thus promoting the precision and personalized development of medical research and clinical treatment. (2) In terms of high-throughput screening, QDs as a marker allows scientists to simultaneously test the effects of multiple compounds or conditions on bone organoids, while the addition of AI greatly improves the efficiency of data processing and quickly identifies potential active ingredients and mechanisms of action. In terms of dose optimization, the fluorescent properties of QDs allow the distribution and concentration of the drug in the bone tissue to be accurately tracked, and AI analyzes these data to help scientists determine the best dose to maximize the therapeutic effect and minimize side effects. In the drug resistance analysis, the behavior of QDS-labeled drug resistance cells was analyzed in detail by AI, revealing key factors in the development of resistance and providing a scientific basis for designing strategies to overcome or prevent resistance. (3) By analyzing labeled bone organoid data, AI technology is able to identify biomarkers and patterns associated with bone disease, thereby predicting the onset and progression of the disease. This predictive ability facilitates early diagnosis and intervention to reduce the risk of disease progression. AI technology can also process the large amount of data generated by QDs labeling, and monitor cellular interactions, dynamic changes in the extracellular matrix, and the distribution of local biochemical factors in real time, providing key information for understanding bone tissue repair and regeneration. Using QDs to track cell behaviors such as migration, proliferation, and differentiation, combined with AI's deep analytical capabilities, can reveal differences in how cells behave in healthy and disease states. This analysis helps identify key factors that influence cell behavior, providing a scientific basis for the development of new therapeutic strategies.

As we all know, the bone microenvironment means complexity [204], and there are still some unsolved mechanisms of action. Researchers can also use AI to study the various mechanisms of action of QDs in BTE, promoting the realization of "two-way love" between bone organoids and QDs.

## 5 Conclusion and future perspectives

BTE aims to research novel materials to improve bone transplantation, promote bone regeneration, and develop alternative therapies to address a wide range of bone diseases. At present, although there are many literature contributions to the application of biomaterials in BTE, there is still a lack of almost perfect materials in BTE. QDs has excellent photoelectric properties, mechanical properties, easy modification and bioconjugation properties, laying a foundation for the preparation of multi-functional nanomaterials in combination with a variety of materials, and is expected to become a new star in bone regeneration scaffolds [205]. In this review, we selectively reviewed and summarized the current research and applications of QDs in the fields of promoting osteogenesis, imaging tracing, drug delivery, antibacterial, phototherapy, and osteoimmunomodulation in BTE, and proposed our own opinions on some of their shortcomings.

Although QDs can promote the development of BTE, it is still rarely seen in the clinic, indicating that there are some challenges that need to be addressed. (1) In clinical application, the most important aspect of the material is its good biocompatibility and security. Some QDs are controversial due to their toxic core components. In early studies, ZnS coating was used to prevent the release of harmful metals [206], but the effect was not significant. The current work should start from two aspects, one is to use more green raw materials to prepare the non-toxic core of QDs, and the other is to try various surface modifications and biological coupling to achieve non-toxic to various cell lines and humans. (2) At the same time, studies have shown that size is an important factor affecting QDs clearance and excretion [207]. To avoid liver and kidney injury, QDs applied to the clinic should be as small as possible (<6 nm, as the filter cutoff size of the kidney is 6–8 nm) to minimize their potentially harmful absorption by liver cells. However, at present, the preparation of QDs is still limited, and the preparation cost of some QDs is high, which can neither achieve the repeatability of characterization nor guarantee the consistency of size. Therefore, if we want to apply QDs to the clinic, we need to find more suitable methods to prepare QDs with consistent size, quality and performance. (3) In addition, the study of QDs in vivo is still insufficient. The microenvironment of human bone is a highly dynamic and complex system, and QDs needs to exhibit adaptations that match the growth rate of new bone, as well as be able to respond to and adapt to biological signals such as growth factors and cytokines that are critical for the growth of new bone. That is, they are required to be able to maintain stability in the changing microenvironment without affecting the normal function of cells and the natural repair process of bone tissue. Second, the long-term stability of QDs is critical, as they need to function continuously in the body without degradation or migration to avoid potential long-term side effects. This is closely related to the surface coupling modification of QDs, which requires researchers to design specific coupling schemes for different situations to meet the needs. In addition, the maintenance of effectiveness of QDs is also a key issue, as they need to maintain their biological activity in the skeletal microenvironment, whether for applications such as fluorescent labeling, drug delivery, or photodynamic therapy. Researchers must address these challenges to further study and promote the use of QDs in BTE clinical trials.

In conclusion, the application of QDs in BTE has broad prospects, and they will greatly promote the development of BTE by providing functions such as high-sensitivity imaging, drug delivery, cell tracking, and phototherapy antibacterial. As QDs technology continues to mature and improve, it is expected to become an important tool in the field of BTE in the future, researchers should face the current opportunities and challenges, and strive to bring more effective, safer, and more personalized treatment options for patients.

## CRediT authorship contribution statement

**Ning Ding:** Writing – original draft. **Fengjin Zhou:** Methodology. **Guangfeng Li:** Investigation. **Hao Shen:** Supervision. **Long Bai:** Conceptualization. **Jiacan Su:** Funding acquisition.

## Declaration of competing interest

We declare that we have no financial and personal relationships with other people or organizations that can inappropriately influence our work, there is no professional or other personal interest of any nature or kind in any product, service, and/or company that could be construed as influencing the position presented in, or the review of, the manuscript entitled, “Quantum Dots for Bone Tissue Engineering”.

## Data Availability

Data will be made available on request.
